# Organ and cell-specific biomarkers of Long-COVID identified with targeted proteomics and machine learning

**DOI:** 10.1186/s10020-023-00610-z

**Published:** 2023-02-21

**Authors:** Maitray A. Patel, Michael J. Knauer, Michael Nicholson, Mark Daley, Logan R. Van Nynatten, Gediminas Cepinskas, Douglas D. Fraser

**Affiliations:** 1 Epidemiology and Biostatistics, Western University, London, ON N6A 3K7 Canada; 2 Pathology and Laboratory Medicine, Western University, London, ON N6A 3K7 Canada; 3 Medicine, Western University, London, ON N6A 3K7 Canada; 4 Computer Science, Western University, London, ON N6A 3K7 Canada; 5 Lawson Health Research Institute, London, ON N6C 2R5 Canada; 6 Medical Biophysics, Western University, London, ON N6A 3K7 Canada; 7 Children’s Health Research Institute, London, ON N6C 4V3 Canada; 8 Pediatrics, Western University, London, ON N6A 3K7 Canada; 9 Clinical Neurological Sciences, Western University, London, ON N6A 3K7 Canada; 10 Physiology and Pharmacology, Western University, London, ON N6A 3K7 Canada; 11 Room C2-C82, London Health Sciences Centre, 800 Commissioners Road East, London, ON N6A 5W9 Canada

**Keywords:** Long-COVID, COVID-19, Targeted proteomics, Machine learning, Organ system, Cell types, Biomarker

## Abstract

**Background:**

Survivors of acute COVID-19 often suffer prolonged, diffuse symptoms post-infection, referred to as “Long-COVID”. A lack of Long-COVID biomarkers and pathophysiological mechanisms limits effective diagnosis, treatment and disease surveillance. We performed targeted proteomics and machine learning analyses to identify novel blood biomarkers of Long-COVID.

**Methods:**

A case–control study comparing the expression of 2925 unique blood proteins in Long-COVID outpatients versus COVID-19 inpatients and healthy control subjects. Targeted proteomics was accomplished with proximity extension assays, and machine learning was used to identify the most important proteins for identifying Long-COVID patients. Organ system and cell type expression patterns were identified with Natural Language Processing (NLP) of the UniProt Knowledgebase.

**Results:**

Machine learning analysis identified 119 relevant proteins for differentiating Long-COVID outpatients (Bonferonni corrected P < 0.01). Protein combinations were narrowed down to two optimal models, with nine and five proteins each, and with both having excellent sensitivity and specificity for Long-COVID status (AUC = 1.00, F1 = 1.00). NLP expression analysis highlighted the diffuse organ system involvement in Long-COVID, as well as the involved cell types, including leukocytes and platelets, as key components associated with Long-COVID.

**Conclusions:**

Proteomic analysis of plasma from Long-COVID patients identified 119 highly relevant proteins and two optimal models with nine and five proteins, respectively. The identified proteins reflected widespread organ and cell type expression. Optimal protein models, as well as individual proteins, hold the potential for accurate diagnosis of Long-COVID and targeted therapeutics.

**Supplementary Information:**

The online version contains supplementary material available at 10.1186/s10020-023-00610-z.

## Introduction

Coronavirus disease 2019 (COVID-19) is a multi-system infection caused by the highly transmissible severe acute respiratory syndrome coronavirus 2 (SARS-CoV-2) (Harrison et al. 2020). SARS-CoV-2 binds to an angiotensin-converting enzyme 2 (ACE2) receptor expressed on the surfaces of many cells for entry (Yuki et al. 2020). COVID-19 severity varies greatly with some experiencing mild symptoms to others experiencing multiorgan failure associated with extracellular matrix changes, impaired immune cell homing and programmed cell death (Iosef et al. 2023).

Approximately 30% of COVID-19 survivors suffer from prolonged, diffuse symptoms including fatigue, dyspnea, neurological symptoms, chest pain and gastrointestinal upset (Nalbandian et al. 2021; Xu et al. 2022; Crook et al. 2021; Xie et al. 2022; Pinto et al. 2022; Nguyen et al. 2022). These prolonged symptoms are termed “Long-COVID”; however, a comprehensive disease classification with participating biomarkers and mechanisms is not defined. Long-COVID symptom presentation is heterogeneous making it challenging to develop clinical models for diagnosis, as well as disease surveillance. The symptoms of Long-COVID are similar to those of patients affected by prolonged SARS, the Middle East respiratory syndrome, and Myalgic Encephalomyelitis/Chronic Fatigue Syndrome (Nalbandian et al. 2021; Crook et al. 2021). Lastly, the timeline of classifying Long-COVID is unclear with some suggesting prolonged symptoms must occur for greater than 4 weeks post-infection and others after 12 weeks (Nalbandian et al. 2021; Nguyen et al. 2022; Maltezou et al. 2021; Raveendran et al. 2021).

A few mechanisms have been proposed to explain the multi-system symptoms of Long-COVID including prolonged hyper-inflammation (Ortelli, et al. 2021; Patterson et al. 2021a; Patterson et al. 2021b), autonomic nervous system disruption (Dani et al. 2021), and persistent thrombosis (Silva Andrade et al. 2021). We recently reported angiogenesis as a key mechanism in Long-COVID outpatients, with the elevation of 14 blood vascular transformation biomarkers (Patel et al. 2022). Identification of accurate Long-COVID-specific biomarkers allows for early disease detection, accurate diagnosis, prognosis and/or targeted therapeutics. Advanced proteomic techniques, such as proximity extension assays [PEA; (Assarsson et al. 2014; Lundberg et al. 2011))] have great potential for an efficient and holistic approach to identifying disease and injury biomarkers (Fraser et al. 2020a; Fraser et al. 2021; Iosef et al. 2023; Van Nynatten et al. 2022).

This study aims to identify blood proteins specific to Long-COVID outpatients, relative to age- and sex-matched acutely ill COVID-19 inpatients and healthy control subjects. Our specific objectives were: (1) to measure a large number of blood proteins with PEA from Long-COVID outpatients, COVID-19 inpatients, and healthy control subjects (2) to determine the relative importance of the proteins in differentiating Long-COVID subjects; and (3) to determine the cell types and organ systems in which the important proteins are expressed.

## Methods

### Study participants and blood sampling

All patients were screened and enrolled from our tertiary care system (London, Ontario, Canada). Both Long-COVID and acutely ill COVID-19 had their COVID-19 status confirmed as part of standard hospital testing by detection of two SARS-CoV-2 viral genes using polymerase chain reaction (CDC 2019-Novel Coronavirus 2019). Long-COVID outpatients had been referred to a specialty clinic based on prolonged, diffuse symptoms. Venous blood was drawn once as part of a larger clinical screen, and excess plasma collected for later research analysis by Pathology and Laboratory Medicine (PaLM). Both Ward and intensive care unit (ICU) patients were enrolled on admission to the hospital. Blood sampling for inpatients began on admission, Ward or ICU Day 1. Daily blood was obtained from critically ill ICU patients via indwelling catheters and if a venipuncture was required, research blood draws were coordinated with a clinically indicated blood draw. In keeping with accepted research phlebotomy protocols for adult patients, blood draws did not exceed maximal volumes (NIH Hrpp 2009). Blood was centrifuged and plasma isolated, aliquoted at 250 µL, and frozen at − 80 °C. All samples remained frozen until use and freeze/thaw cycles were avoided. The healthy control subjects were individuals without disease, acute illness, or prescription medications that were previously banked in the Translational Research Centre, London, ON (Directed by Dr. D.D. Fraser; https://translationalresearchcentre.com/) (Brisson et al. 2012; Gillio-Meina et al. 2013). These latter samples were obtained prior to the emergence of SARS-CoV-2 in our region and therefore, were considered to not have been exposed to the virus.

### Patient demographics, clinical data, and cohort matching

Baseline characteristics for Long-COVID, Ward and, ICU patients were recorded and included age, sex, comorbidities, presenting symptoms, interventions, and laboratory measurements. For Long-COVID patients, we recorded both initial infection variables and clinical variables at follow-up clinic visit. For the latter, we focused on lingering symptoms, laboratory values and interventions. For ICU patients, we included standard illness severity scores, including Multiple Organ Dysfunction Score (MODS) (Priestap et al. 2020) and Sequential Organ Failure Assessment scores (Singer et al. 2016). The PaO_2_ to FiO_2_ ratio and chest radiograph findings were recorded for all ICU patients. We also recorded clinical interventions received during the observation period including the use of antibiotics, antiviral agents, systemic corticosteroids, vasoactive medications, venous thromboembolism prophylaxis, antiplatelet, or anticoagulation treatment, renal replacement therapy, high flow oxygen therapy, and mechanical ventilation (invasive and non-invasive). Final participant groups were constructed by age- and sex-matching Long-COVID outpatients with Ward COVID-19 inpatients, ICU COVID-19 inpatients, and healthy control subjects.

### Proximity extension assay

Plasma was thawed for PEA testing (Olink Proteomics, Sweden) as previously described (Lundberg et al. 2011; Assarsson et al. 2014). Specifically, we measured a total of 3072 plasma proteins in the plasma of Long-COVID, acutely ill COVID-19, and healthy control subjects. The Olink Explore 3072 library consists of multiple panels with some duplicated proteins leading to the measurement of 2925 unique proteins. The PEA was performed in three steps: (1) antibody pairs, labeled with unique DNA oligonucleotides, were attached to their target antigen in plasma; (2) oligonucleotides that were brought into proximity hybridized and were extended by a DNA polymerase; and (3) the newly formed DNA barcode was amplified for high-sensitivity, high-specificity readout with next generation sequencing (NovaSeq Platform; Illumina Inc., San Diego, CA). Data were generated and expressed as relative quantification on the log2 scale of normalized protein expression (NPX) values. Data were converted from log2 scale to normal scale to better represent protein expression. Samples were screened based on quality controls for immunoassay and detection, as well as degree of hemolysis. Following proteomic quality control, all 88 (22 healthy control, 22 Ward COVID-19, 22 ICU COVID-19, and 22 Long-COVID) patients/subjects were deemed suitable for analysis.

### Conventional statistics

Patient baseline clinical characteristics were reported as median (IQRs) for continuous variables and frequency (%) for categorical variables. The individual biomarkers of Long-COVID outpatients were compared to a combined group of healthy controls, Ward COVID-19 inpatients, and ICU COVID-19 inpatients, using a Mann–Whitney U Test. A Kruskal–Wallis H-test for independent samples followed by a pairwise posthoc Dunn test was also conducted for the optimal models. A Bonferroni correction was applied to avoid multiple comparison complications, with only Bonferroni-corrected P-values being reported and those with a P < 0.01 were considered to be statistically significant.

### Machine learning

For machine learning, a Random Forest classifier based on decision trees was used to classify the Long-COVID cohort in comparison to a combined cohort of acutely ill COVID-19 ward/ICU inpatients and healthy control subjects by their biomarkers. The Boruta feature reduction algorithm was used to identify the most important biomarkers (Kursa and Rudnicki 2010). The Boruta algorithm is based on Random Forest classifiers and individually compares each biomarker to randomly generated data to determine if the biomarker is better at classifying than chance. The results from the Boruta feature reduction identified the most relevant biomarkers for classifying Long-COVID.

The following steps were undertaken to conduct a conservative analysis that mitigates concerns of relatively small sample sizes and overfitting due to Boruta feature reduction being based on Random Forest classifiers. First, the data was split into a feature reduction dataset (70%) and a testing dataset (30%), stratified by subject groups. The Boruta algorithm was run on the feature reduction dataset to determine the most relevant features. A reduced dataset was created from the testing dataset and only contained the most relevant features. The reduced dataset was then used for the classification of Long-COVID. To reduce overfitting and maintain a conservative model, three-fold cross-validation with a Random Forest of 10 trees and a maximum depth of three was used (Tang et al. 2018).

To prepare an optimal model, recursive feature elimination (RFE) was used. As a Random Forest is a set of decision trees, we were able to interrogate this collection of trees to identify the features that have the highest predictive value (viz., those features that frequently appear near the top of the decision tree). Based on this characteristic, RFE starts with the reduced dataset, fits a Random Forest classifier and determines the importance rankings. The algorithm then drops the least important feature and repeats the process until only 10 features are remaining. Due to the randomness in the algorithm and Random Forest models, 10,000 runs of RFE were conducted. Those features that were in the top 10 for more than a specified threshold of the 10,000 runs were determined to be the optimal features. The specified threshold is determined after the inspection of the RFE results. An optimal dataset containing only these optimal features was generated from the reduced dataset. The same classification process used for the reduced dataset was used on the optimal dataset.

Receiver operating characteristic (ROC) curves using Logistic Regression were conducted to determine the sensitivity and specificity of individual molecules for predicting Long-COVID status in comparison to healthy controls and COVID-19 patients. Area-under-the-curve (AUC) was calculated as an aggregate measure of protein performance across all possible classification thresholds (Bradley 1997). Precision and Recall were determined, including their combined metric (F1 score), which was calculated as the harmonic mean. A high F1 score indicated that both, Precision and Recall were high. The biomarker data was visualized with a nonlinear dimensionality reduction on the full, reduced, and optimal datasets using the t-distributed stochastic nearest neighbour embedding (t-SNE) algorithm. t-SNE assumes that the ‘optimal’ representation of the data lies on a manifold with complex geometry, but a low dimension, embedded in the full-dimensional space of the raw data (Van der Maaten and Hinton 2008).

A pairwise comparison, using cosine similarity, was conducted to determine the similarity between subjects across the selected biomarkers (Jambu 1991). As such, subjects similar across their selected biomarker profile have a score closer to 1, while dissimilar subjects have a score closer to 0. The analysis was done with data Min–Max scaled between 0 and 1 and the cosine similarities were visualized using a heatmap. The machine learning analysis was conducted using Python version 3.9.7 and Scikit-Learn version 1.0.2 (Pedregosa et al. 2011).

### Natural language processing

Exploratory expression analysis was also conducted to determine physiological areas of interest in Long-COVID subjects. Protein expression tissue specificity was parsed from UniProt Knowledgebase using the UniProt website REST API (Bateman et al. 2021). The tissue specificity was unstructured text on the expression at the mRNA or protein level in cells or tissues gathered manually by experts. The expression information was processed by Natural Language Processing (NLP) using the Stanza python package implemented with spaCy (Python v. 3.10.4; spaCy v. 3.3.1; spaCy-Stanza v. 1.0.2; negspaCy v. 1.0.3) (Zhang et al. 2021a; Qi et al. 2020; Honnibal et al. 2020). An NLP named-entity recognition (NER) pipeline was configured with the MIMIC package for preprocessing, negation detection, and the pretrained Stanza BioNLP13CG Biomedical model. The negation detection was done using the NegEx-based negspaCy implementation with a modified English clinical term set to filter negative expression terms. Although the BioNLP13CG biomedical model was based on Cancer Genetics and publicly available PubMed abstracts, in comparison to the other Stanza models, it provided the most granular entity classification, including anatomical system, organ, tissue, multi-level tissue, and cell type entities. The detected organ and cell type entities were manually classified into keyword-based groups separately. The manual expression curation process relies on existing literature and is not easily structured into specific organ systems. To include the maximum expression information in the analysis, the organ, tissue, multi-tissue, and anatomical system entity types were combined and manually sorted into organ systems. The frequency of the keyword-based categories with respect to the relevant proteins was determined to identify physiological patterns of expression.

## Results

A total of 4 age- and sex-matched groups were included consisting of Long-COVID outpatients (median years old = 61; IQR = 21; n = 22), Ward COVID-19 inpatients (median years old = 60; IQR = 22; n = 22), ICU COVID-19 inpatients (median years old = 58; IQR = 18; n = 22) and healthy control subjects (median years old = 59; IQR = 16; n = 22). There were no significant differences concerning age (Kruskal–Wallis H-test, P = 0.9880) and sex (Chi-Square, P = 1.000) between the 4 cohorts. Baseline demographic characteristics, comorbidities, laboratory measurements, interventions, and chest x-ray findings of Long-COVID outpatients and Ward/ICU COVID-19 inpatients, are reported in Tables 1 and 2 respectively. Long-COVID outpatients had a single blood draw at their clinic visit, whereas blood from Ward and ICU COVID-19 inpatients was drawn on day 1 of hospital admission. Long-COVID patients had normal lymphocyte measurements (2.0; normal range 1.0–4.0 × 10^9^), while both Ward and ICU COVID-19 patients had abnormally low values (P < 0.0001). The mortality rates for Ward and ICU COVID-19 inpatients were 9.1% and 45.5%, respectively.

**Table 1:** Long-COVID outpatient demographics and clinical data

Initial infection variable	Outpatients (n = 22)
Age (yrs), median (IQR)	61.0 (20.5)
Male sex, no. (%)	12 (54.5)
Diagnostic test: PCR, serology, no. (%)	22 (100.0)
Vaccination status at infection, no. (%)	2 (9.1)
Hospitalization, no. (%)	
Ward	7 (30.4)
ICU	1 (4.3)
Comorbidities, no. (%)	
Diabetes	6 (27.3)
Hypertension	8 (36.4)
Coronary artery/heart disease	2 (9.1)
Chronic/congestive heart failure	0 (0.0)
Chronic kidney disease	0 (0.0)
Cancer	1 (4.5)
COPD	0 (0.0)
Asthma	4 (18.2)
Presenting symptoms at infection, no. (%)	
Fever	16 (72.7)
Cough	17 (77.3)
Anosmia/Ageusia	13 (59.1)
Pharyngitis	8 (36.4)
Headache	14 (63.6)
Confusion/Memory	2 (9.1)
Myalgias	13 (59.1)
Dyspnea	16 (72.7)
Chest pain	8 (36.4)
Nausea/Vomiting/Diarrhea	11 (50.0)
Interventions at infection, no. (%)	
Steroids	6 (27.3)
Remdesivir	0 (0.0)
Tocilizumab	1 (4.5)
Long-COVID Clinic Variables	
Follow up, days from infection onset, median (IQR)	101.5 (45.5)
Lingering symptoms at follow up, no. (%)	
Respiratory	16 (72.7)
Cardiovascular	6 (27.3)
Neurologic	8 (36.4)
Musculoskeletal	0 (0.0)
Gastro-Intestinal	3 (13.6)
Psychiatric	1 (4.5)
Cutaneous	0 (0.0)
Balance	0 (0.0)
Chest pain	4 (18.2)
Concentration	0 (0.0)
Cough	2 (9.1)
Dyspnea	16 (72.7)
Fatigue	11 (50.0)
Headache	2 (9.1)
Low mood	1 (4.5)
Anxiety	1 (4.5)
Memory	6 (27.3)
Nausea	1 (4.5)
Palpitations	1 (4.5)
Paresthesia	1 (4.5)
Smell/taste	2 (9.1)
Word finding	1 (4.5)
Non-specific	11 (50.0)
Laboratories at follow up, median (IQR)	
White blood cell count	7.1 (1.9)
Neutrophils	4.5 (1.5)
Lymphocytes	2.0 (0.7)
Hemoglobin	139.5 (24.8)
Platelets	239.5 (64.2)
C-Reactive Protein (CRP)	1.8 (3.5)
Ferritin	76.0 (118.8)
Lactate Dehydrogenase (LDH)	206.0 (39.0)
Alanine Aminotransferase (ALT)	20.0 (11.2)
Interventions at follow up, no. (%)	
Budesonide	1 (4.5)
Anticoagulant	1 (4.5)
Budesonide/Formoterol	10 (45.5)
Salbutamol	3 (13.6)
Furosemide	1 (4.5)
Nasal spray	2 (9.1)
Oxygen	2 (9.1)
Physiotherapy	4 (18.2)
None	8 (36.4)

**Table 2:** Acutely ill COVID-19 inpatient demographics and clinical data

Variable	Ward Inpatients (n = 22)	ICU Inpatients (n = 22)
Age (yrs), median (IQR)	60.0 (21.5)	58.0 (17.5)
Male sex, no. (%)	12 (54.5)	12 (54.5)
Weight (kg), median (IQR)	84.8 (14.8)	90.0 (28.3)
Height (cm), median (IQR)	169.0 (9.2)	170.0 (9.0)
BMI, median (IQR)	28.6 (5.6)	30.5 (7.6)
MODS, median (IQR)	–	5.0 (1.0)
SOFA Score, median (IQR)	–	5.5 (5.8)
Comorbidities, no. (%)		
Diabetes	4 (18.2)	10 (45.5)
Hypertension	9 (40.9)	9 (40.9)
Coronary artery/heart disease	1 (4.5)	2 (9.1)
Chronic/congestive heart failure	0 (0.0)	0 (0.0)
Chronic kidney disease	1 (4.5)	2 (9.1)
Cancer	3 (13.6)	2 (9.1)
COPD	0 (0.0)	1 (4.5)
Presenting symptoms, no. (%)		
Fever	18 (81.8)	–
Cough	18 (81.8)	–
Anosmia/Ageusia	4 (18.2)	–
Pharyngitis	4 (18.2)	–
Headache	3 (13.6)	–
Myalgias	14 (63.6)	–
Dyspnea	20 (90.9)	–
Chest pain	3 (13.6)	–
Nausea/Vomiting/Diarrhea	9 (40.9)	–
Pulmonary pathology, no. (%)		
Unilateral pneumonia	–	1 (4.5)
Bilateral pneumonia	21 (95.5)	20 (90.9)
Interstitial infiltrates/R effusion	–	1 (4.5)
Laboratories, median (IQR)		
Hemoglobin	129.5 (23.0)	118.5 (29.8)
White Blood Cell count	6.8 (4.9)	8.8 (7.9)
Neutrophils	5.8 (3.9)	7.5 (7.4)
Lymphocytes	0.8 (0.7)	0.7 (0.6)
Platelets	210.0 (68.5)	220.0 (143.5)
Creatinine	69.5 (25.5)	79.5 (86.2)
International Normalized Ratio	1.0 (0.1)	1.2 (0.1)
Lactate	1.7 (0.9)	1.2 (0.8)
Partial thromboplastin time (PTT)	–	26.5 (5.0)
PaO_2_/FiO_2_ Ratio	–	128.5 (62.5)
Interventions, no. (%)		
Renal replacement therapy	0 (0.0)	5 (22.7)
High-flow nasal cannula	13 (59.1)	15 (68.2)
Non-invasive mechanical ventilation	1 (4.5)	6 (27.3)
Invasive mechanical ventilation	2 (9.1)	20 (90.9)
Extracorporeal membrane oxygenation	0 (0.0)	1 (4.5)
Tocilizumab	2 (9.1)	0 (0.0)
Steroids	21 (95.5)	14 (63.6)
Vasoactive medications	2 (10.0)	18 (81.8)
Antibiotics	22 (100.0)	22 (100.0)
Anti-virals	4 (18.2)	3 (13.6)
Antiplatelet	4 (18.2)	17 (77.3)
Anticoagulation	22 (100.0)	21 (95.5)
Outcomes		
Days, median (IQR)	9.0 (6.8)	15.5 (15.0)
Died, no. (%)	2 (9.1)	10 (45.5)

**Fig. 1:**
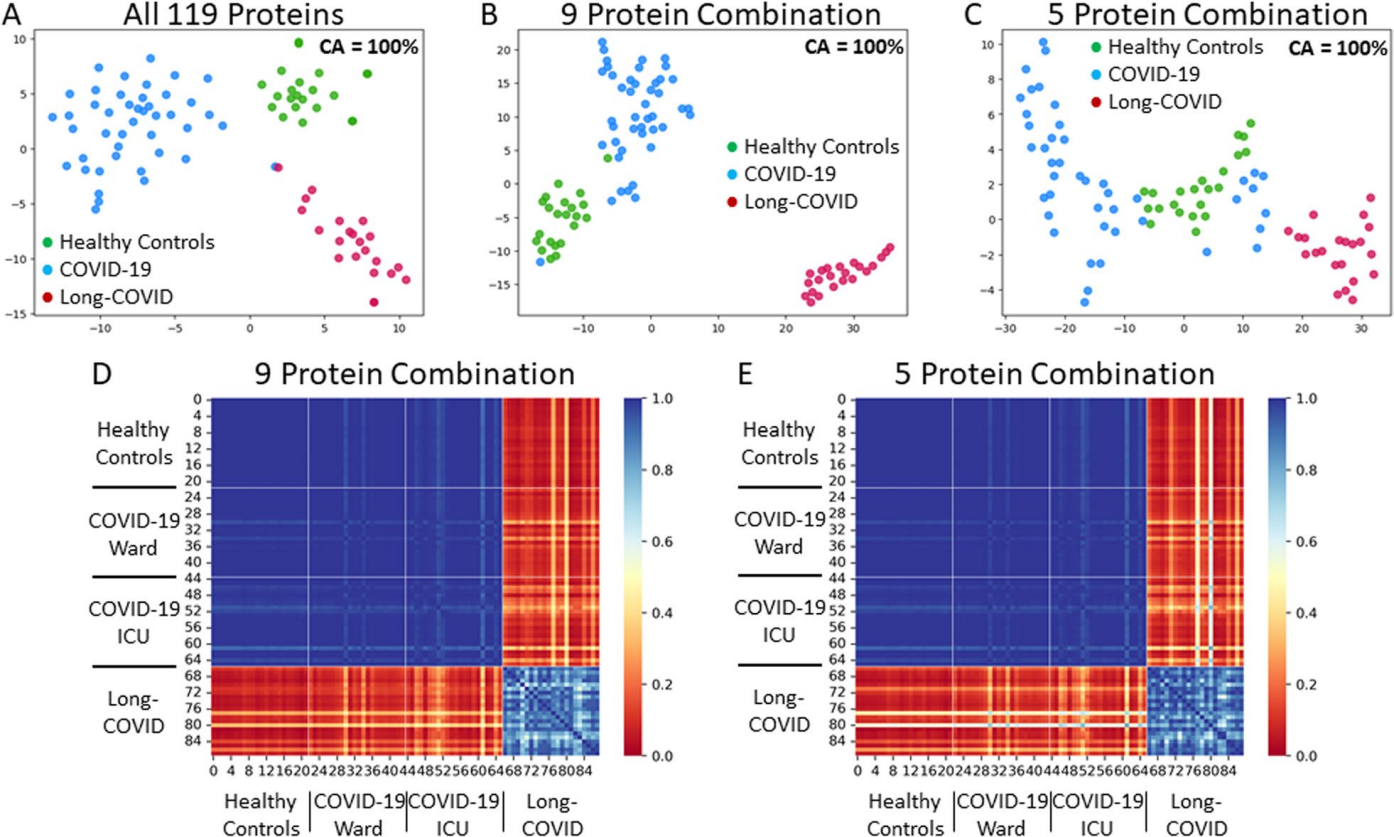
Identification of important blood proteins in Long-COVID outpatients.** A** Subjects plotted in two dimensions, following t-SNE dimensionality reduction of all 119 important proteins determined by Boruta feature reduction, shows cluster separation of Long-COVID outpatients from acutely ill COVID-19 ward/ICU inpatients and healthy control subjects. **B** Subjects plotted in two dimensions, following t-SNE dimensionality reduction of top 9 important proteins determined by Recursive Feature Selection with 50% threshold, shows separation cluster of Long-COVID outpatients from acutely ill COVID-19 ward/ICU inpatients and healthy control subjects **C** Subjects plotted in two-dimensions, following t-SNE dimensionality reduction of top 5 important proteins determined by Recursive Feature Selection with 80% threshold, shows separation cluster of Long-COVID outpatients from acutely ill COVID-19 ward/ICU inpatients and healthy control subjects with some mixing **D** A heatmap demonstrated the pairwise cosine similarity between cohort’s protein profiles for top 9 proteins. Greater cosine similarity measure between subjects indicates similar protein profiles while smaller measure indicates large differences between profiles (distance was pseudocolored on the bar scale). The protein profile of Long-COVID outpatients is distinctively different from all other cohorts. **E** A heatmap demonstrated the pairwise cosine similarity between cohort’s protein profiles with respect to top 5 proteins. Greater cosine similarity measure between subjects indicates similar protein profiles while smaller measure indicates large differences between profiles (distance was pseudocolored on the bar scale). The protein profile of Long-COVID outpatients is distinctively different from all other cohorts

A total of 2,925 unique biomarkers were identified following the removal of duplicates. After a Boruta feature reduction, 119 plasma biomarkers were identified and found to be useful in classifying Long-COVID outpatients when compared to a combined cohort of acutely ill COVID-19 inpatients and healthy control subjects (Additional file 1: Table S1). All 119 relevant biomarkers were significantly different between Long-COVID subjects and the other subjects as calculated by the Mann–Whitney U test with Bonferroni multiple-comparison correction (corrected individual P < 0.0001, significant P < 0.01). Of the 119 biomarkers, only 10 exhibited decreased expression (FRZB, FN1, CKMT1A_CKMT1B, HS6ST1, BMP6, ANGPTL2, IFNLR1, C1QA, DRAXIN, and ADAMTSL4). Each of the 119 relevant biomarkers had excellent individual classification ability with AUCs ranging between 0.91 and 1.00. Using the 119 relevant blood biomarkers, a t-SNE plot illustrated that Long-COVID patients were easily separable from acutely ill COVID-19 inpatients and healthy control subjects (Fig. 1A; classification accuracy 100%, AUC 1.00, F1 1.00).

**Fig. 2:**
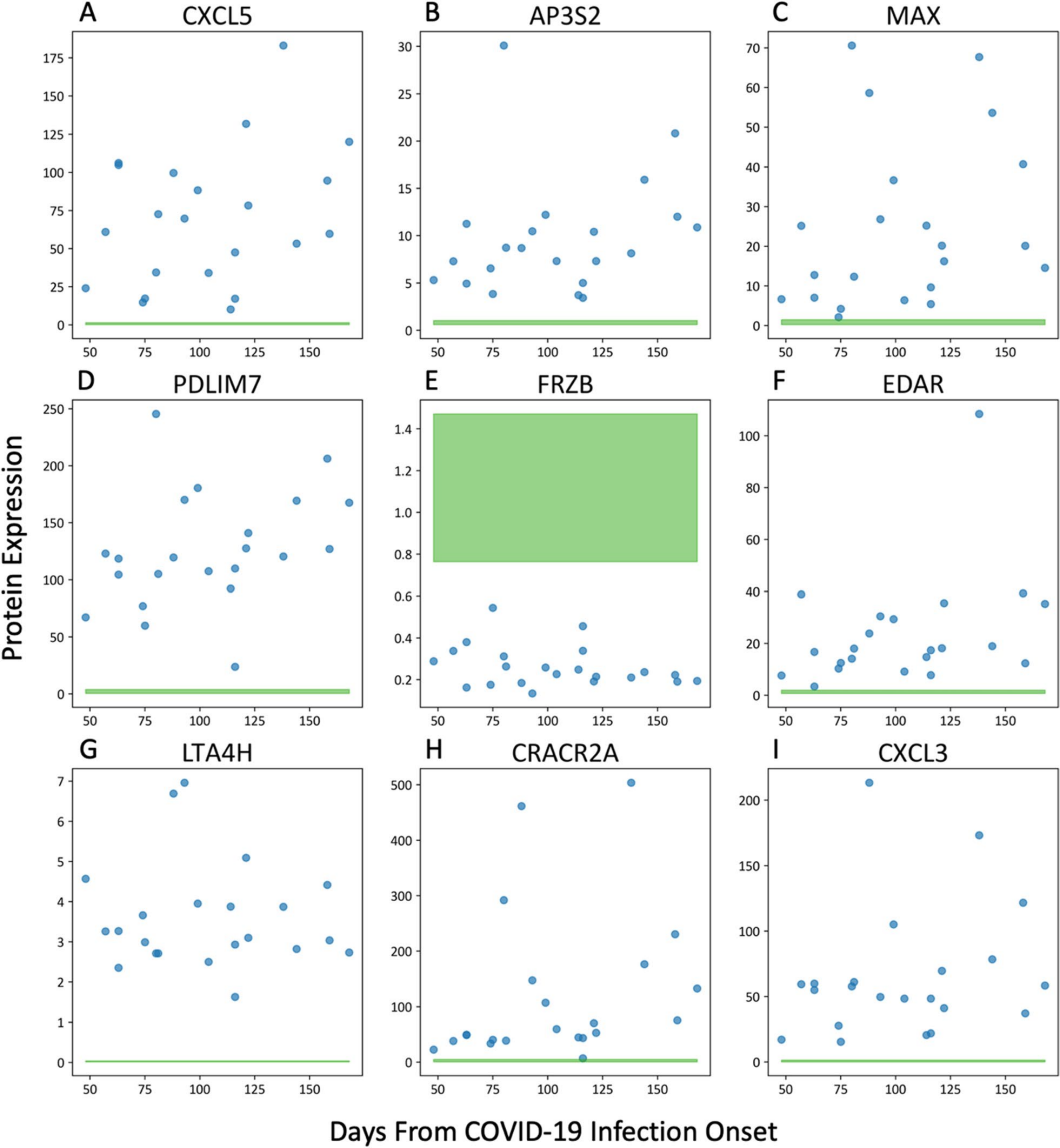
Protein Expression of Optimal 9 Proteins in Long-COVID. Blue points are Long-COVID outpatient measurements; green filled area represents 5th percentile to 95th percentile protein expression range of healthy control subjects. **A–D, F–I** Plots demonstrating elevated protein expression in Long-COVID compared to healthy controls versus time after acute infection for CXCL5, AP3S2, MAX, PDLIM7, EDAR, LTA4h, CRACR2A, CXCL3. **E** A plot demonstrating decreased FRZB expression in Long-COVID compared to healthy controls versus time after acute infection

Recursive feature elimination was used to determine two sets of optimal proteins, one with a threshold of 50% and another with a threshold of 80% (Additional file 1: Fig. S1). The threshold represents the percentage of runs, out of 10,000 RFE repetitions, that a particular protein was in the top 10 reduced proteins. With the threshold of 50%, an optimal set of nine proteins (CXCL5, AP3S2, MAX, PDLIM7, EDAR, LTA4H, CRACR2A, CXCL3, FRZB) was determined from the 119 relevant proteins. A t-SNE plot based on the nine optimal biomarkers showcases a distinct separation between Long-COVID outpatients and the acutely ill COVID-19 inpatients and healthy control subjects (Fig. 1B; classification accuracy 100%, AUC 1.00, F1 1.00). With a threshold of 80%, an optimal set of five proteins (CXCL5, AP3S2, MAX, PDLIM7, FRZB) was determined from the 119 relevant proteins. A t-SNE plot based on the five optimal biomarkers showcases a distinct separation between the Long-COVID outpatients and the acutely ill COVID-19 inpatients and healthy control subjects (Fig. 1C; classification accuracy 100%, AUC 1.00, F1 1.00). All nine of the optimal biomarkers had excellent individual classification ability with an AUC of 1.00, except for CRACR2A which had an AUC of 0.97 (Additional file 1: Tables S1 and S2). All of the nine optimal proteins were significantly elevated in Long-COVID outpatients, other than FRZB which was significantly decreased in Long-COVID outpatients (Fig. 2; Additional file 1: Table S2). The functions of the optimal nine proteins were described (Additional file 1: Table S3). Confounding variables, such as steroid administration, were excluded via correlation analysis between patient/subject variables and protein expression (data not shown).

Pairwise cosine similarity between all subjects was calculated to compare the cohorts in terms of holistic nine and five optimal protein profiles, presented in Fig. 1D, E, respectively. For both nine and five optimal protein sets, the protein profile between the healthy control subjects and acutely ill COVID-19 inpatients was homogeneous. The Long-COVID outpatients were relatively less homogeneous but clearly distinct from the other cohorts.

**Fig. 3:**
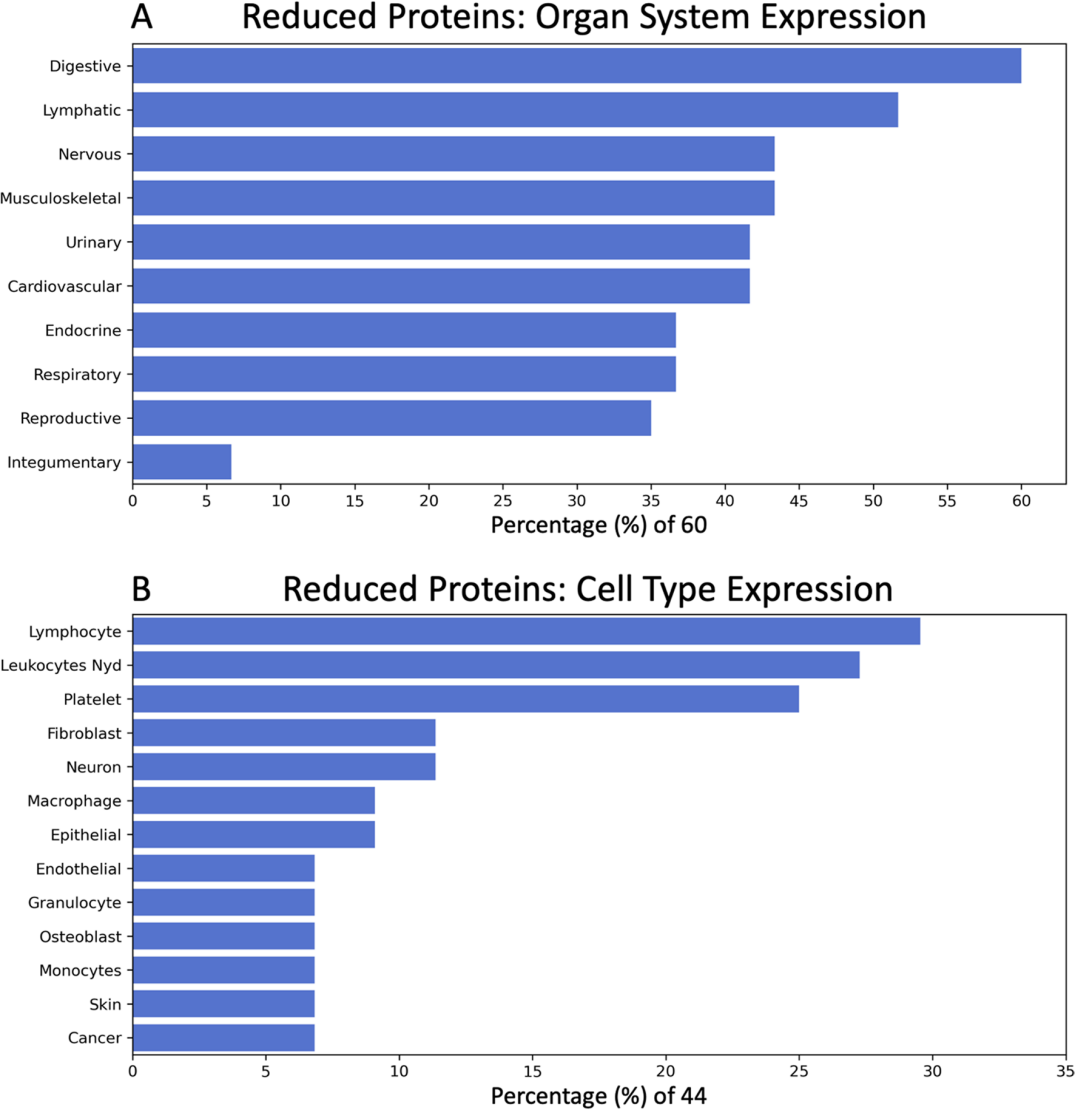
Frequency of protein expression in major organs/body systems and cell type. **A** A bar plot demonstrating the percentage of proteins that are expressed in specific major organs and body systems determined by Natural Language Processing. There were total of 60 proteins out of the 119 proteins (50%) with UniProt organ system expression information. The organ system classification combines NLP identified organs, tissue, multi-level tissue and anatomical system entities. **B** A bar plot demonstrating the percentage of proteins that are expressed in specific cell types determined by Natural Language Processing. There were total of 44 proteins out of the 119 proteins (37%) with UniProt cell type expression information. Only those cell types with percentages greater than 5% are shown for visualization clarity

Named-entity recognition was conducted on the tissue expression information provided by the UniProt Knowledgebase. Out of the 119 reduced proteins, 60 (50.4%) had organ expression information (Additional file 1: Table S4) and 44 (37.0%) had cell type expression information (Additional file 1: Table S5). The percentage of the 60 molecules that are expressed in specific organ systems and the percentage of the 44 molecules that are expressed in specific cell types are presented in Fig. 3A, B respectively. The leading organ system based on the number of changed proteins was the digestive system. Analyses of cell type expression demonstrated that the number of changed proteins was greatest in lymphocytes/leukocytes not yet determined.

## Discussion

In this study, we measured the expression of 2925 blood proteins using targeted proteomics for age- and sex-matched Long-COVID outpatients, acutely ill COVID-19 inpatients (Ward and ICU) and healthy control subjects. Using machine learning algorithms, we identified 119 important proteins that differentiate Long-COVID outpatients from other cohorts, indicating a unique protein profile. Two optimal models, with a subset of nine and five proteins, accurately differentiated Long-COVID patients from acutely ill COVID-19 inpatients and healthy control subjects (classification accuracy of 100%, AUC of 1.00, F1 1.00). Organ and cell type expressions were examined with NLP of the UniProt Knowledgebase.

Our patient cohorts were similar to those reported in earlier studies with regard to demographics, comorbidities and clinical presentation. The Long-COVID outpatients suffered diffuse symptoms across multiple organ systems, such as fatigue, post-exertional malaise, anosmia and cognitive dysfunction (Carfì et al. 2020; Davis et al. 2021). With regards to acutely ill COVID-19 patients, they were also similar to those reported in earlier cohorts (Myers et al. 2020; Bhatraju, et al. 2020; Zhou et al. 2020; Wu et al. 2020), and demonstrated significant inflammatory and thrombotic mechanisms (Fraser et al. 2020a; Fraser et al. 2020b; Fraser et al. 2020c; Gill et al. 2020), as well as microvascular injury (Fraser et al. 2020d).

Our study identified 119 proteins that differentiated Long-COVID outpatients from acutely ill COVID-19 inpatients and healthy control subjects. Each of the 119 proteins was significantly different in Long-COVID outpatients, as compared to other cohorts, and had individual AUCs ranging from 0.91 to 1.00. The models with a reduced number of biomarkers were created to provide specific research targets for future studies assessing disease specificity, diagnostics and understanding of Long-COVID pathophysiology. The first optimal model contained nine proteins: CXCL5, AP3S2, MAX, PDLIM7, FRZB, EDAR, LTA4H, CRACR2A, and CXCL3. The optimal second model is a subset of the first with five proteins: CXCL5, AP3S2, MAX, PDLIM7, and FRZB. Each of the optimal models demonstrated excellent classification and AUC, as well as precision and recall. Each of the nine optimal proteins was significantly different in Long-COVID outpatient when compared pair-wise to the other cohorts**.** Of the nine proteins, eight had increased expression and one decreased. The functions of the optimal nine proteins varied widely and appropriately corroborated that Long-COVID is a multifaceted condition in which multiple systems are affected.

NLP, a subset of artificial intelligence, was used to identify organ and cell type expression patterns of the significant 119 proteins. Expert curated expression information from UniProt Knowledgebase was parsed using NLP to identify key cell types, organs, tissues, major tissue systems and anatomical systems. Parsed information from the latter four expression categories was combined to represent the organ system to which the molecules were being expressed within. Of the 119 proteins, 60 had organ system expression information and 44 had cell type expression information. Overall, other than the integumentary system, the reduced proteins are highly expressed in all other organ systems, corroborating again the multi-system symptom presentation in Long-COVID patients.

The digestive system had the highest number of significant proteins with altered expression. This finding was consistent with a significant gut biome change identified in Long-COVID patients when compared to both controls and recovered COVID-19 patients without Long-COVID symptoms (Liu et al. 2022). Gastrointestinal and digestive symptoms, including vomiting, nausea and diarrhea, have been reported in Long-COVID patients (Groff et al. 2021; Huang et al. 2021). Mutations in AP3S2, one of the optimal five proteins, were associated with type 2 diabetes mellitus (Kazakova, et al. 2017; Kooner et al. 2011; Mohlke and Boehnke 2015) and may be related to the hypothesized increase in type 2 diabetes mellitus due to COVID-19 (Rubino et al. 2020). CES3 played an important role in adipocyte differentiation and promoted lipid storage (Dominguez et al. 2014). In CES3 knockout mice, insulin sensitivity and glucose tolerance improved (Wei et al. 2010). In animal models, VPS37A changed intracellular receptor localization such that overexpression of VPS37A resulted in decreased blood glucose levels (Sekar 2022) and in Long-COVID may be a protective effect to counteract the AP3S2 and CES3 overexpression. Overexpression of SRC is involved with colon cancer and often results in metastasis via its signalling pathways (Chen et al. 2014).

The lymphatic system appeared to be highly affected in Long-COVID as more than 50% of the 60 significant proteins had expression in lymphatic organs. Of the 44 proteins with cell type expression information, lymphocytes and leukocytes not yet determined were the two most common cell types. Consistent with protein expression, autopsies of critically ill COVID-19 patients have revealed changes to the structure of the spleen and lymph nodes (Liu et al. 2020). CRACR2A and CXCL3 were both linked to immune cell activation and may indicate an ongoing immune response in Long-COVID outpatients. CRACR2A participated in T-cell activation and functional CRACR2A changes were linked to immunodeficiency disorders (Srikanth, et al. 2016; Wu, et al. 2021; Notarangelo et al. 2020). CXCL3 was linked to activating neutrophils, basophils, eosinophils, monocytes, smooth muscle cells, and lymphocytes (Laing and Secombes 2004). Three of the optimal nine proteins, CXCL5, LTA4H, and CXCL3, as well as CCL5, CCL11, CCL13, CCL17, and CCL26 from the 119 proteins were pro-inflammatory (Laing and Secombes 2004; Mendez-Enriquez and García-Zepeda 2013; Fourie 2009; Chang et al. 1994; Larose et al. 2015; Soria and Ben-Baruch 2008; Ponath et al. 1996). CCL3 and CCL5 were reported previously to be elevated in Long-COVID patients (Patterson et al. 2021b). Several immune cell receptors were also a part of the top 119 proteins including CD226, CD84, CD40LG, and CD69. These inflammatory proteins were all significantly elevated in Long-COVID patients when compared to healthy control subjects and acutely ill COVID-19 subjects.

Long-COVID appeared to highly impact the nervous system with symptoms often including headaches, fatigue, and brain fog (Raveendran et al. 2021; Ortelli, et al. 2021). The NLP expression analysis showed that a large number of proteins are highly expressed in the nervous system, particularly in neurons. FRZB, AP3S2, and MAX were not only part of the optimal model, but were also linked to neurological conditions. FRZB was linked to defects in sensory innervation and spinal innervation (John et al. 2012), and decreased FRZB expression was associated with increased neuronal development (Jang et al. 2013). AP3S2 was a small chain of the Adaptor-related protein complex 3 (AP-3). AP-3 subunit defects lead to severe neurological abnormalities including neurodevelopmental delays, intellectual disability and seizures (Guardia et al. 2018). MAX mutations were associated with hereditary pheochromocytoma, a neural crest cell-based neuroendocrine tumour in the adrenal medulla (Comino-Méndez et al. 2011; Burnichon et al. 2012). Beyond the panel of optimal proteins, PLXNB3, APP, and BDNF were also associated with neurological conditions. PLXNB3, overexpressed in our Long-COVID outpatients has been previously linked to COVID-19 (Yaşar et al. 2021); it was shown to stimulate neurite outgrowth in mice and was also associated with verbal performance and brain white matter volume in humans (Hartwig et al. 2005; Rujescu et al. 2007). Overexpression of APP was either a protective response leading to cell health and growth, or detrimental with increased Aß accumulation and decreased dendritic synapses (Hoe et al. 2012; O'Brien and Wong 2011). BDNF upregulation was shown to increase proliferation and differentiation of neural stem cells (Lee et al. 2016).

Survivors of acute COVID-19 were at an increased risk of developing cardiovascular disorders including ischemic heart disease, inflammatory heart disease, dysrhythmias, and thrombotic disorders (Xie et al. 2022). Vascular endothelial injury, angiogenesis and thrombosis were associated with acute COVID-19 pathophysiology (Ackermann, et al. 2020; Fraser et al. 2020e). Similarly, coagulation and inflammation were associated with Long COVID (Nalbandian et al. 2021; Pretorius, et al. 2021). We previously reported significant elevations in 14 vascular transformation biomarkers, including ANGPT1 and SELP, which are also a part of the top 119 proteins in this study (Patel et al. 2022). ANGPT1 has vascular protective effects while ANGPTL2 promoted angiogenesis (Thorin-Trescases and Thorin 2014; Brindle et al. 2006); however, in Long-COVID subjects, the ANGPT1/ANGPT2 ratio is dramatically increased indicating that the angiopoietin system is associated with vascular protection. Overexpression of PEAR1 was associated with decreased proliferation of microvascular endothelial cells further corroborating active vascular protection (Zhan et al. 2020). Several pro-coagulation factors were in the top 119 proteins including GP5, GP6, and STX8 (Moog et al. 2001; Golebiewska et al. 2015). GP5 and GP6 are involved in platelet adhesion and aggregation (Moog et al. 2001; Veninga et al. 2022) and STX8 is involved in platelet granule secretion, aggregation and thrombus stability (Golebiewska et al. 2015). Overexpression of GP6 was linked to large, reactive juvenile platelets (Veninga et al. 2022) and surface presentation of GP6-dimers is linked to thrombotic disorders (Induruwa et al. 2022). CASP2, part of the top 119 proteins, has previously been identified to be upregulated in COVID-19 and cardiomyopathy (Lee et al. 2021). Overall, Long-COVID pathophysiology may show active vascular protection or healing, as well as increased coagulation.

Long-COVID subjects often report prolonged respiratory symptoms with the most common being dyspnea (Pinto et al. 2022). Several of the 119 top proteins were associated with the respiratory system including EDAR, CCL17, EREG, GTPBP2, and DRG2. EDAR was previously identified to be altered in COVID-19 patients with lung epithelium injury (D’Agnillo, et al. 2021). Elevated CCL17 is associated with various pulmonary conditions including idiopathic pulmonary fibrosis, asthma and COPD and cigarette smoke-induced pulmonary inflammation (Yogo et al. 2009; Machida et al. 2022; Staples et al. 2012). CCL17 was also noted to be an important biomarker for eosinophilic disorders including differentiating eosinophilic pneumonia from acute lung injury (Miyazaki et al. 2007; Catherine and Roufosse 2021). EREG was involved in cell proliferation and differentiation of airway epithelial cells (Riese and Cullum 2014). Overexpression of EREG, GTPBP2, and DRG2 was linked to tumor growth in non-small cell lung cancer (Jie et al. 2021; Sunaga et al. 2008; Hong et al. 2018). FRZB is involved in the WNT/ ß-catenin pathway and serves as a WNT antagonist (Dale 1998). The WNT/ß-catenin pathway has been linked to various conditions including sepsis and inflammation, and was hypothesized to be involved in COVID-19 pulmonary fibrosis (Nusse and Clevers 2017; Zhang et al. 2021b; Satu et al. 2021). Increased FRZB was observed in acutely ill severe COVID-19 (Teng et al. 2021), but depressed in our Long-COVID outpatients.

SARS-CoV-2 utilizes the ACE2 receptor for cellular entry via spike protein binding, and the ACE2 receptor is a critical component of the renin–angiotensin–aldosterone system (RAAS) that is involved in renal, vascular, and myocardial functions (Martínez-Salazar et al. 2022). Downregulation of the ACE2 receptor during acute infection may lead to RAAS dysregulation, including electrolyte, cardiovascular and pulmonary dysfunction in Long-COVID (Sui et al. 2021; Lei et al. 2021; Pedrosa Maria et al. 2021; Cooper et al. 2021; Mandal et al. 2021). Reduced ACE2 function is linked to activation of the des-Arg^9^ bradykinin (DABK)/bradykinin receptor B1 (BKB1R), potentially increasing neutrophil infiltration and release of proinflammatory cytokines such as CXCL5 (Abassi et al. 2021; Sodhi et al. 2018). This latter mechanism is consistent with the elevated CXCL5 in Long-COVID outpatients demonstrated by our proteomic study.

Our study has identified 119 key proteins and developed optimal models with nine and five proteins; however, our study has several limitations. First, we included an equal, yet conservative number of matched subjects within each group. Nonetheless, we ensured robust analysis via non-parametric statistics and conservative machine learning parameters. With regards to the latter, potential overfitting due to a nested feature selection was reduced by performing 10,000 repetitions of RFE, testing the optimal models’ performance on the unused test dataset, and conducting no hyper-parameter optimization. Second, our data showed altered protein expression in Long-COVID outpatients; however, we did not have longitudinal samples from each subject to determine protein changes over time with eventual normalization. Third, our analysis showcased models that differentiated Long-COVID outpatients from acutely ill COVID-19 inpatients and healthy control subjects; however, we cannot confirm these protein models were distinct from other pathologies. Cross-identity concerns can be mitigated by using a multiple protein combination, together with recognition of diffuse symptoms post-SAR-CoV-2 infection (PCR-positive acute illness or nucleocapsid antibody testing). A combined model would decrease the likelihood that other pathologies alter the same biomarkers, and in the same temporal manner, as Long-COVID. Fourth, previously collected healthy control samples were used to verify the absence of a prior SARS-CoV-2 infection, as our latest attempts to collect healthy control samples without previous infection or a recent vaccination were insufficient. While it is possible that some proteins in plasma can be susceptible to both storage duration and temperature, strict sample processing and storage protocols were followed. Lastly, the pre-trained NLP model cannot identify organ and cell type expression for proteins without information in UniProt Knowledgebase. Despite these caveats, and given the scarcity of knowledge on Long-COVID pathophysiology, our exploratory investigation provides valuable insights.

## Conclusion

The lack of Long-COVID-specific biomarkers limits accurate diagnosis and treatment, as well as disease surveillance. In this study, we identified 119 key proteins and developed two accurate models with nine and five proteins, respectively. These exploratory results provide valuable insight for future studies investigating Long-COVID pathophysiological mechanisms, diagnosis, and therapeutics.

## Supplementary Information

**Additional file 1. Table S1.** Classification Accuracy (Random Forest) and ROC Area-Under-the-Curve Analyses.   **Table S2.** Expression of the Top 9 Proteins in Specific Cohorts. **Table S3.** Function of the Top 9 Proteins.  **Table S4.** Expression NLP Categories by Organ System for the Top 119 Proteins. **Table S5.** Expression NLP Categories by Cell Type for the  Top 119 Proteins. **Fig. S1.** Recursive Feature Selection of 119 Protein Results after 10000 Runs

## Data Availability

The datasets generated and/or analysed during the current study are available from the corresponding author on reasonable request.

## Abbreviations

Aß: Amyloid-beta
ACE2: Angiotensin-converting enzyme 2
ANGPT1: Angiopoietin-1
ANGPT2: Angiopoietin-2
AP3: Adaptor-related protein complex 3
AP3S2: Adaptor-related protein complex 3 subunit sigma 2
APP: Amyloid Beta Precursor Protein
AUC: Area-under-the-curve
BDNF: Brain-derived neurotrophic factor
CCL5: C-C motif chemokine 5
CCL11: C-C motif chemokine 11
CCL13: C-C motif chemokine 13
CCL17: C-C motif chemokine 17
CCL26: C-C motif chemokine 26
CES3: Carboxylesterase 3
COVID-19: Coronavirus disease 2019
CRACR2A: Calcium release activated channel regulator 2A
CXCL3: C-X-C Motif Chemokine Ligand 3
CXCL5: C-X-C Motif Chemokine Ligand 5
DRG2: Developmentally-Regulated GTP-binding protein 2
EDAR: Ectodysplasin A Receptor
EREG: Epiregulin
FiO_2_: Fractional inspired oxygen
FRZB: Frizzled related protein
GP5: Glycoprotein 5
GP6: Glycoprotein 6
GTPBP2: GTP-binding protein 2
ICU: Intensive care unit
LTA4H: Leukotriene A4 hydrolase
MAX: MYC associated factor X
MERS: Middle East Respiratory Syndrome
ME/CFS: Myalgic Encephalomyelitis/Chronic Fatigue Syndrome
MODS: Multiple organ dysfunction score
NER: Named entity recognition
NLP: Natural language processing
NPX: Normalized protein expression
PaLM: Pathology and Laboratory Medicine
PaO_2_: Partial Pressure of Oxygen
PDLIM7: PDZ and LIM Domain 7
PEA: Proximity extension assay
PLXNB3: Plexin B3
RAAS: Renin–Angiotensin–Aldosterone System
RFE: Recursive Feature Elimination
ROC: Receiver Operating Characteristic
SARS: Severe Acute Respiratory Syndrome
SARS-CoV-2: Severe Acute Respiratory Syndrome Coronavirus 2
SELP: P-Selectin
STX8: Syntaxin 8
t-SNE: T-distributed Stochastic Nearest Neighbor Embedding
VPS37A: Vacuolar Protein Sorting-Associated Protein 37A
WNT: Wingless and Int-1
