# Supplementary material for: Organ and cell-specific biomarkers of Long-COVID identified with targeted proteomics and machine learning
Source: Mol Med. 2023 Feb 21;29:26. doi: 10.1186/s10020-023-00610-z (PMC9942653; doi:10.1186/s10020-023-00610-z)
Supplement: Supplementary file 1 — Additional file 1. Table S1. Classification Accuracy (Random Forest) and ROC Area-Under-the-Curve Analyses. Table S2. Expression of the Top 9 Proteins in Specific Cohorts. Table S3. Function of the Top 9 Proteins. Table S4. Expression NLP Categories by Organ System for the Top 119 Proteins. Table S5. Expression NLP Categories by Cell Type for the Top 119 Proteins. Fig. S1. Recursive Feature Selection of 119 Protein Results after 10000 Runs [file 10020_2023_610_MOESM1_ESM.docx]

**Additional file 1: Table S1. Classification Accuracy (Random Forest) and ROC Area-Under-the-Curve Analyses.**

| **UniProt** | **Assay** | **Long-COVID** | **Healthy and COVID** | **ROC Logistic AUC** | **Bonferroni Corrected P Value** | **Feature Importance %** |
| --- | --- | --- | --- | --- | --- | --- |
| Q86WV1 | SKAP1 | 60.5 (34.4-75.6) | 0.6 (0.5-0.8) | 1 | 3.24E-10 | 1.14 |
| P52564 | MAP2K6 | 68.9 (60.4-82.0) | 3.3 (1.6-5.2) | 1 | 3.24E-10 | 1.12 |
| Q9H7M9 | VSIR | 42.4 (28.9-73.5) | 1.4 (1.0-1.8) | 1 | 3.24E-10 | 1.12 |
| Q8TF64 | GIPC3 | 50.8 (34.5-66.6) | 3.3 (1.4-7.1) | 1 | 3.24E-10 | 1.1 |
| P23560 | BDNF | 10.9 (8.4-15.3) | 0.5 (0.3-0.9) | 1 | 3.24E-10 | 1.08 |
| P68106 | FKBP1B | 46.7 (28.0-67.3) | 1.2 (0.9-1.5) | 1 | 3.24E-10 | 1.06 |
| P05067 | APP | 20.2 (10.5-25.3) | 1.4 (1.0-1.7) | 1 | 3.24E-10 | 1.06 |
| P19876 | CXCL3 | 56.5 (38.2-67.5) | 1.5 (0.7-3.0) | 1 | 3.24E-10 | 1.04 |
| Q92765 | FRZB | 0.2 (0.2-0.3) | 1.5 (1.1-2.0) | 1 | 3.24E-10 | 1.02 |
| P42830 | CXCL5 | 65.3 (34.2-98.4) | 0.8 (0.5-1.1) | 1 | 3.24E-10 | 1 |
| Q9HCN6 | GP6 | 36.7 (25.7-45.7) | 1.5 (0.9-2.5) | 1 | 3.24E-10 | 0.96 |
| P20340 | RAB6A | 22.7 (8.0-42.6) | 0.3 (0.3-0.5) | 1 | 3.24E-10 | 0.96 |
| P09960 | LTA4H | 3.2 (2.8-3.9) | 0.0 (0.0-0.1) | 1 | 3.24E-10 | 0.96 |
| P02775 | PPBP | 16.7 (10.7-19.8) | 0.5 (0.2-1.1) | 1 | 3.24E-10 | 0.94 |
| Q96A25 | TMEM106A | 11.9 (9.0-23.8) | 1.6 (1.0-2.0) | 1 | 3.24E-10 | 0.94 |
| P16109 | SELP | 8.7 (6.4-11.9) | 1.1 (0.8-1.4) | 1 | 3.24E-10 | 0.94 |
| P02751 | FN1 | 0.2 (0.2-0.2) | 1.3 (1.0-1.6) | 1 | 3.24E-10 | 0.92 |
| P12532 | CKMT1A_CKMT1B | 0.1 (0.0-0.1) | 2.4 (1.8-3.1) | 1 | 3.24E-10 | 0.92 |
| Q86YW5 | TREML1 | 15.7 (10.8-21.1) | 1.1 (0.8-1.6) | 1 | 3.24E-10 | 0.9 |
| O60243 | HS6ST1 | 0.2 (0.1-0.2) | 1.4 (0.8-2.7) | 1 | 3.24E-10 | 0.9 |
| Q9HD42 | CHMP1A | 7.7 (5.9-8.7) | 1.7 (0.7-2.5) | 1 | 3.24E-10 | 0.88 |
| P29965 | CD40LG | 20.8 (10.1-25.3) | 1.1 (0.7-1.8) | 1 | 3.24E-10 | 0.88 |
| O14944 | EREG | 40.8 (19.0-61.1) | 0.7 (0.5-1.1) | 1 | 3.24E-10 | 0.88 |
| Q9Y2X7 | GIT1 | 23.0 (15.1-32.4) | 3.6 (1.7-5.2) | 1 | 3.24E-10 | 0.86 |
| P42575 | CASP2 | 32.5 (18.6-53.1) | 2.8 (1.8-4.0) | 1 | 3.24E-10 | 0.86 |
| P01133 | EGF | 53.1 (31.3-65.4) | 1.2 (0.6-2.4) | 1 | 3.24E-10 | 0.84 |
| Q9H0P0 | NT5C3A | 80.7 (70.5-113.6) | 4.8 (2.0-9.3) | 1 | 3.24E-10 | 0.82 |
| P22004 | BMP6 | 0.2 (0.1-0.3) | 1.3 (0.9-1.4) | 1 | 3.24E-10 | 0.76 |
| P40197 | GP5 | 2.2 (2.0-2.4) | 0.6 (0.4-0.9) | 1 | 3.24E-10 | 0.74 |
| P12931 | SRC | 72.0 (53.7-109.6) | 4.2 (2.0-7.4) | 1 | 3.24E-10 | 0.72 |
| Q07108 | CD69 | 84.8 (59.9-120.9) | 5.2 (2.4-9.0) | 1 | 3.24E-10 | 0.7 |
| Q9UBW5 | BIN2 | 100.2 (52.2-203.9) | 3.8 (2.3-6.5) | 1 | 3.24E-10 | 0.66 |
| P01024 | C3 | 1.5 (1.2-1.9) | 0.2 (0.1-0.2) | 1 | 3.24E-10 | 0.62 |
| Q9Y6A5 | TACC3 | 188.9 (131.0-306.6) | 6.0 (2.8-11.1) | 1 | 3.47E-10 | 1.16 |
| Q9ULL4 | PLXNB3 | 6.5 (4.6-9.9) | 1.0 (0.8-1.4) | 1 | 3.47E-10 | 1.08 |
| Q9Y2Y0 | ARL2BP | 15.3 (11.5-29.9) | 1.3 (0.9-1.7) | 1 | 3.47E-10 | 1 |
| Q9UHD8 | SEPTIN9 | 32.2 (24.4-66.8) | 1.8 (1.3-2.4) | 1 | 3.47E-10 | 0.86 |
| Q9NUY8 | TBC1D23 | 54.9 (36.9-82.7) | 3.5 (1.9-6.4) | 1 | 3.47E-10 | 0.82 |
| O75351 | VPS4B | 17.2 (11.9-25.6) | 1.8 (1.0-2.5) | 1 | 3.47E-10 | 0.76 |
| P40818 | USP8 | 12.6 (6.6-21.7) | 0.5 (0.4-0.9) | 1 | 3.47E-10 | 0.74 |
| P55957 | BID | 6.6 (4.5-10.1) | 0.4 (0.2-0.6) | 1 | 3.72E-10 | 1.22 |
| Q9UJU6 | DBNL | 68.4 (50.7-98.4) | 4.2 (1.7-6.6) | 1 | 3.72E-10 | 1.1 |
| Q99616 | CCL13 | 23.0 (20.1-30.9) | 2.2 (1.6-3.2) | 1 | 3.72E-10 | 1.06 |
| Q9UKW4 | VAV3 | 241.4 (101.6-622.6) | 4.4 (2.1-9.2) | 1 | 3.72E-10 | 0.98 |
| Q5VY43 | PEAR1 | 2.2 (2.0-2.8) | 1.0 (0.9-1.2) | 1 | 3.72E-10 | 0.98 |
| P13501 | CCL5 | 12.3 (8.6-19.4) | 1.1 (0.3-1.8) | 1 | 3.72E-10 | 0.96 |
| Q14790 | CASP8 | 26.4 (17.7-36.5) | 0.6 (0.3-0.9) | 1 | 3.72E-10 | 0.94 |
| O00194 | RAB27B | 47.2 (41.1-60.8) | 12.0 (3.9-23.0) | 1 | 3.72E-10 | 0.9 |
| Q13976 | PRKG1 | 199.8 (123.7-557.2) | 8.8 (3.5-17.3) | 1 | 3.72E-10 | 0.88 |
| Q9UIB8 | CD84 | 3.5 (3.0-3.8) | 1.2 (0.9-1.5) | 1 | 3.72E-10 | 0.86 |
| Q9BQS7 | HEPH | 1.1 (0.9-1.1) | 0.4 (0.3-0.4) | 1 | 3.72E-10 | 0.86 |
| Q6P589 | TNFAIP8L2 | 14.3 (7.8-20.0) | 0.9 (0.7-1.3) | 1 | 3.72E-10 | 0.82 |
| Q8TE58 | ADAMTS15 | 1.2 (0.9-1.7) | 5.6 (4.1-8.0) | 1 | 3.72E-10 | 0.78 |
| P55273 | CDKN2D | 32.7 (23.9-37.6) | 1.8 (1.0-3.1) | 1 | 3.72E-10 | 0.74 |
| Q15389 | ANGPT1 | 14.3 (10.0-16.8) | 1.1 (0.5-1.9) | 1 | 3.72E-10 | 0.72 |
| O75167 | PHACTR2 | 23.9 (14.4-38.0) | 2.0 (1.1-3.2) | 1 | 3.72E-10 | 0.6 |
| Q9UNE0 | EDAR | 17.7 (12.4-30.2) | 1.1 (0.8-1.5) | 1 | 3.98E-10 | 1.34 |
| O95644 | NFATC1 | 19.2 (9.3-22.6) | 1.6 (1.0-2.3) | 1 | 3.98E-10 | 1.06 |
| Q13561 | DCTN2 | 111.7 (59.5-196.2) | 3.5 (2.1-6.3) | 1 | 3.98E-10 | 0.88 |
| Q92609 | TBC1D5 | 80.6 (57.7-111.7) | 6.7 (3.3-12.6) | 1 | 3.98E-10 | 0.86 |
| Q16206 | ENOX2 | 5.9 (3.4-12.0) | 1.3 (1.0-1.6) | 1 | 3.98E-10 | 0.8 |
| Q08AG7 | MZT1 | 16.2 (10.8-39.5) | 1.6 (1.3-2.2) | 1 | 3.98E-10 | 0.7 |
| Q12765 | SCRN1 | 25.7 (18.8-35.4) | 1.5 (1.0-2.5) | 1 | 4.27E-10 | 1.1 |
| P04085 | PDGFA | 11.4 (8.1-13.1) | 0.7 (0.3-1.2) | 0.99 | 4.27E-10 | 0.96 |
| P23743 | DGKA | 43.8 (26.5-81.0) | 2.8 (1.9-4.5) | 1 | 4.27E-10 | 0.94 |
| Q15762 | CD226 | 6.6 (4.6-8.4) | 0.9 (0.7-1.5) | 1 | 4.27E-10 | 0.92 |
| P53990 | IST1 | 18.6 (14.0-26.0) | 1.7 (0.8-2.7) | 1 | 4.27E-10 | 0.88 |
| Q13576 | IQGAP2 | 67.0 (47.0-105.1) | 2.3 (1.3-4.1) | 1 | 4.27E-10 | 0.86 |
| O60496 | DOK2 | 39.8 (20.9-57.3) | 3.5 (1.7-5.3) | 1 | 4.57E-10 | 1.12 |
| Q92783 | STAM | 11.5 (8.7-18.3) | 1.5 (1.0-1.9) | 1 | 4.57E-10 | 1.12 |
| Q9NR12 | PDLIM7 | 120.1 (104.7-160.9) | 4.2 (2.0-9.7) | 1 | 4.57E-10 | 0.96 |
| P55039 | DRG2 | 9.3 (4.6-12.8) | 1.2 (0.9-1.7) | 1 | 4.57E-10 | 0.88 |
| Q6ZRY4 | RBPMS2 | 167.1 (141.3-190.4) | 15.5 (4.1-33.5) | 1 | 4.57E-10 | 0.86 |
| Q9BX10 | GTPBP2 | 55.3 (36.0-122.8) | 2.6 (1.5-4.3) | 0.99 | 4.57E-10 | 0.8 |
| P80162 | CXCL6 | 9.2 (7.9-11.7) | 1.1 (0.8-1.6) | 1 | 4.57E-10 | 0.74 |
| Q9UKU9 | ANGPTL2 | 0.3 (0.2-0.5) | 1.4 (1.1-1.9) | 0.99 | 4.89E-10 | 1.04 |
| P42574 | CASP3 | 23.3 (18.1-44.7) | 4.5 (1.8-7.2) | 0.99 | 4.89E-10 | 0.96 |
| Q8NEZ2 | VPS37A | 9.9 (6.8-13.2) | 1.5 (1.0-2.0) | 1 | 4.89E-10 | 0.96 |
| P49137 | MAPKAPK2 | 13.0 (9.6-20.7) | 2.0 (1.4-3.3) | 0.99 | 4.89E-10 | 0.9 |
| O60884 | DNAJA2 | 35.3 (27.5-45.2) | 4.3 (1.6-6.4) | 1 | 4.89E-10 | 0.9 |
| Q15276 | RABEP1 | 24.6 (18.5-62.9) | 2.6 (1.7-4.4) | 1 | 4.89E-10 | 0.84 |
| P13807 | GYS1 | 16.2 (7.9-23.2) | 0.6 (0.4-1.2) | 1 | 4.89E-10 | 0.62 |
| Q8N1Q1 | CA13 | 62.1 (38.6-96.7) | 3.1 (1.5-4.5) | 0.99 | 5.23E-10 | 0.8 |
| Q5SW79 | CEP170 | 48.5 (33.9-54.8) | 7.5 (2.6-10.9) | 1 | 5.24E-10 | 0.98 |
| P09104 | ENO2 | 8.4 (4.9-16.1) | 1.6 (1.1-2.5) | 0.99 | 5.24E-10 | 0.46 |
| Q8IU57 | IFNLR1 | 0.3 (0.3-0.4) | 1.4 (1.0-1.9) | 0.99 | 5.24E-10 | 0.36 |
| O43665 | RGS10 | 6.3 (4.1-8.5) | 1.5 (1.3-1.7) | 1 | 5.60E-10 | 1.26 |
| P61244 | MAX | 18.2 (7.7-34.2) | 1.1 (0.7-1.7) | 1 | 5.60E-10 | 1.02 |
| O75190 | DNAJB6 | 47.4 (35.1-88.9) | 3.3 (1.9-5.3) | 1 | 5.60E-10 | 0.98 |
| P59780 | AP3S2 | 8.4 (5.6-11.2) | 1.2 (0.8-1.5) | 1 | 5.60E-10 | 0.9 |
| Q9NWM8 | FKBP14 | 14.0 (6.9-33.7) | 1.4 (0.9-1.8) | 1 | 5.60E-10 | 0.74 |
| Q96RT1 | ERBIN | 21.5 (14.0-31.2) | 2.2 (1.3-3.5) | 1 | 5.60E-10 | 0.68 |
| O00161 | SNAP23 | 12.0 (7.3-14.6) | 0.8 (0.4-1.5) | 1 | 6.00E-10 | 0.98 |
| O94986 | CEP152 | 13.3 (8.2-35.2) | 1.6 (0.9-2.3) | 1 | 6.00E-10 | 0.8 |
| Q9BV40 | VAMP8 | 36.2 (26.4-46.7) | 5.5 (2.4-8.2) | 1 | 6.42E-10 | 0.58 |
| P51671 | CCL11 | 2.8 (2.5-3.0) | 1.1 (0.8-1.4) | 1 | 6.87E-10 | 0.94 |
| P78352 | DLG4 | 39.5 (23.6-122.8) | 2.3 (1.4-4.2) | 0.99 | 6.87E-10 | 0.84 |
| Q96IU4 | ABHD14B | 15.6 (11.3-20.3) | 0.6 (0.4-0.8) | 1 | 6.87E-10 | 0.7 |
| P05154 | SERPINA5 | 1.6 (1.1-1.7) | 0.4 (0.3-0.6) | 0.99 | 6.87E-10 | 0.46 |
| P02776 | PF4 | 8.7 (6.1-9.7) | 0.7 (0.5-1.3) | 0.98 | 8.41E-10 | 0.54 |
| Q9UDT6 | CLIP2 | 47.9 (32.5-65.9) | 7.1 (3.0-13.2) | 0.98 | 9.00E-10 | 0.92 |
| Q92583 | CCL17 | 8.5 (5.5-12.2) | 0.6 (0.4-1.2) | 0.99 | 9.62E-10 | 0.86 |
| Q9P2T1 | GMPR2 | 30.9 (16.4-58.2) | 3.4 (1.9-5.3) | 1 | 9.62E-10 | 0.8 |
| P02745 | C1QA | 0.7 (0.6-0.8) | 1.6 (1.2-2.0) | 0.98 | 9.62E-10 | 0.22 |
| Q9HD26 | GOPC | 15.5 (9.9-24.1) | 3.1 (1.7-4.5) | 1 | 1.18E-09 | 0.64 |
| Q9Y258 | CCL26 | 13.6 (9.6-20.7) | 0.9 (0.6-1.4) | 0.98 | 1.26E-09 | 0.92 |
| P41227 | NAA10 | 30.9 (17.2-36.9) | 4.1 (1.8-6.0) | 1 | 1.35E-09 | 0.42 |
| Q8N129 | CNPY4 | 9.5 (5.9-16.5) | 1.4 (1.0-2.2) | 1 | 1.54E-09 | 0.56 |
| P30405 | PPIF | 3.9 (2.6-14.7) | 1.3 (1.1-1.5) | 0.97 | 1.54E-09 | 0.28 |
| Q9UNK0 | STX8 | 18.1 (12.7-21.6) | 2.0 (1.1-3.3) | 0.99 | 1.64E-09 | 0.72 |
| Q8NBI3 | DRAXIN | 0.4 (0.3-0.6) | 1.6 (1.1-2.3) | 0.99 | 1.88E-09 | 0.32 |
| Q6UY14 | ADAMTSL4 | 0.8 (0.6-1.0) | 4.4 (1.9-7.7) | 0.99 | 1.88E-09 | 0.1 |
| P31431 | SDC4 | 3.3 (2.6-4.3) | 0.3 (0.1-0.5) | 0.99 | 2.01E-09 | 0.64 |
| Q9NRY6 | PLSCR3 | 21.7 (16.1-31.8) | 3.4 (2.2-4.7) | 0.99 | 2.14E-09 | 0.24 |
| Q9BSW2 | CRACR2A | 56.2 (41.0-143.9) | 6.1 (2.6-9.9) | 0.97 | 2.29E-09 | 0.92 |
| Q99683 | MAP3K5 | 72.3 (38.9-115.7) | 5.9 (3.0-11.1) | 0.96 | 3.87E-09 | 0.9 |
| P09341 | CXCL1 | 24.9 (18.3-34.1) | 2.3 (1.2-4.0) | 0.97 | 4.13E-09 | 0.1 |
| Q15797 | SMAD1 | 4.3 (2.8-7.9) | 1.3 (0.9-1.6) | 0.97 | 5.02E-09 | 1.16 |
| Q6UWW8 | CES3 | 0.3 (0.2-0.5) | 0.1 (0.1-0.1) | 0.91 | 7.19E-07 | 0.06 |

Note: Long-COVID, Long-COVID outpatients (n=22); Healthy and COVID, health control subjects (n=22), acutely Ward COVID inpatients (n=22), acutely ill ICU COVID inpatients (n=22). Mann-Whitney U test with Bonferroni multiple comparisons correction

**Additional file 1: Table S2. Expression of the Top 9 Proteins in Specific Cohorts.**

| **UniProt** | **Protein** | **Long-COVID** | **ICU** | **Ward** | **Healthy** | **P Value** | **Change** |
| --- | --- | --- | --- | --- | --- | --- | --- |
| P42830 | CXCL5 | 65.3 (34.2-98.4) | 0.9 (0.5-1.2) | 0.9 (0.6-1.4) | 0.6 (0.3-0.8) | <0.001 | ↑ |
| P59780 | AP3S2 | 8.4 (5.6-11.2) | 1.4 (1.2-2.0) | 1.4 (1.2-1.5) | 0.7 (0.7-0.8) | <0.001 | ↑ |
| P61244 | MAX | 18.2 (7.7-34.2) | 1.4 (0.8-2.0) | 1.4 (0.8-1.9) | 0.8 (0.6-1.1) | <0.001 | ↑ |
| Q9NR12 | PDLIM7 | 120.1 (104.7-160.9) | 5.8 (3.7-18.1) | 8.7 (5.2-11.6) | 1.2 (0.8-2.1) | <0.001 | ↑ |
| Q9UNE0 | EDAR | 17.7 (12.4-30.2) | 1.5 (0.9-2.2) | 0.9 (0.7-1.2) | 1.1 (0.9-1.3) | <0.001 | ↑ |
| P09960 | LTA4H | 3.2 (2.8-3.9) | 0.1 (0.1-0.1) | 0.1 (0.0-0.1) | 0.0 (0.0-0.0) | <0.001 | ↑ |
| Q9BSW2 | CRACR2A | 56.2 (41.0-143.9) | 8.2 (6.3-11.7) | 9.1 (6.2-12.7) | 1.9 (1.2-2.5) | <0.001 | ↑ |
| P19876 | CXCL3 | 56.5 (38.2-67.5) | 2.4 (1.5-3.1) | 2.7 (1.5-4.2) | 0.6 (0.3-0.8) | <0.001 | ↑ |
| Q92765 | FRZB | 0.2 (0.2-0.3) | 1.9 (1.5-2.1) | 1.8 (1.5-2.0) | 1.2 (1.0-1.3) | <0.001 | ↓ |

Note: P Value (Bonferroni Adjusted Post-hoc Dunn Test) represents the largest Long-COVID pairwise comparison with Healthy, Ward COVID-19 and ICU COVID-19. Change represents the change Long-COVID relative to acute COVID and healthy controls.

**Additional file 1: Table S3. Function of the Top 9 Proteins**

| **UniProt** | **Assay** | **OLINK Panel** | **Function** |
| --- | --- | --- | --- |
| P42830 | CXCL5 | Cardiometabolic | CXC Motif Chemokine Ligand 5 aka Epithelial-derived Neutrophil-activating peptide 78 (CXCL5, ENA-78), a small molecule peptide, interacts with GPCRs to attract neutrophils during inflammation processes (1). It has also been identified to participate in angiogenesis, tumor growth, and metastasis (2, 3). CXCL5 has been linked to various cancers as a promotor (2, 4, 5) and in inflammatory diseases (6, 7). |
| P59780 | AP3S2 | Neurology II | Adapter-related protein complex 3 subunit sigma-2 (AP3S2) is a small chain of the clathrin-based Adapter-related heterotetramer protein complex 3 (AP-3) (8). Two forms of AP-3 with different chain variantions exist, a ubiquitous AP-3 and a brain-specific AP-3, both of which have AP3S2 chains (9). AP-3 in mammals is linked to the lysosome and lysosome-related organelles as well as neurotransmitter release mechanisms (8, 10). SNPs in the AP3S2 have been associated with type 2 diabetes mellitus in Chinese and South Asian populations (11-13). Defects in other neuron AP-3 subunits have shown to lead to severe neurological abnormalities including neurodevelopmental delays, intellectual disability and seizures (14). |
| P61244 | MAX | Neurology | MYC -associated factor X (MAX) is a transcription regulator and is critical to oncoprotein MYC function (15) which is associated with cell growth. As such MAX is a potential target for cancer drugs to inhibit MYC function (16). Mutations of MAX are proposed to cause hereditary pheochromocytoma, a type of neuroendocrine tumor consisting of neural crest cells localized in the adrenal medulla (17, 18). MAX is also associated with regulating clock gene expression, part of the circadian clock (19). |
| Q9NR12 | PDLIM7 | Inflammation | The PDZ and LIM domain protein 7 (PDLIM7, Enigma) binds to protein kinases using the LIM domain and actin filaments via the PDZ domain (20). PDLIM7 in mise is primarily found in actin-rich structures like the heart and vascular smooth muscle (21). PDLIM7 has been connected to vascular and heart development (22, 23) and is also linked to skeletal muscle development (24, 25). |
| Q9UNE0 | EDAR | Inflammation | Ectodysplasin A receptor (EDAR) interacts with ectodysplasin A as a cell surface receptor that is critical for cell signaling and developmental pathways (26). EDAR has been associated with ectodermal dysplasia resulting in errors or differences in hair, teeth, and exocrine gland development (26-30). |
| P09960 | LTA4H | Oncology | Leukotriene-A4 hydrolase (LTA4H) has two functions: converting LTA4 into LTB4, a neutrophil chemoattractant, and aminopeptidase activity (31). The proinflammatory role of LTA4H through LTB4 activity allows for LTA4H to be an anti-inflammatory target (32, 33). LTA4H is also proposed as a cancer therapeutic target as LTA4H as it is found to be overexpressed in cancer (34). |
| Q9BSW2 | CRACR2A | Oncology | Calcium release-activated channel regulator 2A (CRACR2A) is a conserved protein expressed in T cells (35). CRACR2A participates in T cell activation and regulation of endocytic traffic via dynein (36). Changes in CRACR2A functioning have been linked to immunodeficiency disorders (37, 38). |
| P19876 | CXCL3 | Inflammation | CXC Motif Chemokine Ligand 3 (CXCL3) is part of the CXC chemokine family (39). CXCL3 may play a role in acute inflammation as it is noted to activate neutrophils, basophils, eosinophils, monocytes, smooth muscle cells, and lymphocytes (40). CXCL3 has been shown to facilitate adipogenesis (41) and plays a role in various cancers (42-44). |
| Q92765 | FRZB | Neurology | Frizzled related protein 3 (FRZB; SFRP3) has a domain similar to the Wnt-binding region of frizzled transmembrane receptors that play an important role in skeletal development (45). FRZB is a Wnt antagonist and functions by sequestering Xwnt-8 (46). Decreased FRZB expression in adult mice dentate gyrus granule neurons is also linked to increased neuron development (47). In mice, FRZB expression in the olfactory nerve layer is important for olfactory axon targeting (48). FRZB has been associated with soft tissue sarcomas in an anti-tumor capacity (49). There is conflicting evidence regarding the relationship between FRZB and bone mineral density/osteoarthritis (50-53). |

**Additional file 1: Table S4. Expression NLP Categories by Organ System for the Top 119 Proteins**

| **Organ System** | **Proteins** | **Keywords** |
| --- | --- | --- |

| Cardiovascular | FN1, BDNF, MAP2K6, APP, FKBP1B, FRZB, CASP2, TBC1D23, PEAR1, DRG2, VPS37A, CASP3, ANGPTL2, DNAJB6, ERBIN, MAX, SERPINA5, ABHD14B, GMPR2, CCL26, STX8, ADAMTSL4, PLSCR3, MAP3K5, SMAD1 | abdominal aorta, aorta, aorta extracellular, aortic, aortic intima, aortic valves, arteries, arteriolar tree, artery, ascending aorta, atria, atrium, atrium cardiomyocytes, blood, blood vessel walls, blood vessels, capillaries, capillary endothelium, cardiac, cardiac, cardiac atria, cardiac muscle, cardiac muscles, cardiovascular, blood vessels, coronary, coronary arteries, coronary artery, coronary artery smooth muscle, dermal blood vessels, ductus arteriosus, fetal heart, heart, heart muscle, heart muscle, heart spleen, heart ventricle, heart ventricles, hearts, inter-ventricular septum, large arteries, large vessels, lateral ventricle, mammary artery, myocardium, myocardium, periosteum, right atrium, right ventricle, skin blood vessels, small capillaries, small vessel endothelium, small vessels, stromal vascular, system vessels, thoracic aorta, umbilical cord artery, vasa vasorum, vascular, vascular, vascular capillary network, vascular endothelium, vascular smooth muscle, vascular structure, vascular structures, vascular system, vascular-rich organs, vasculature, vasculature, vein, veins, ventricle, ventricles, ventricular, ventricular trabeculae, vessel wall, vessels |
| --- | --- | --- |
| Digestive | GIPC3, CASP2, BIN2, SRC, FRZB, EREG, APP, MAP2K6, EGF, NT5C3A, TBC1D23, PLXNB3, CCL13, ADAMTS15, BID, CASP8, PEAR1, DRG2, PDLIM7, DOK2, VPS37A, ANGPTL2, CASP3, CA13, DNAJB6, ERBIN, MAX, SERPINA5, ABHD14B, CCL17, GMPR2, STX8, ADAMTSL4, PLSCR3, MAP3K5, CES3 | adult colon epithelium, adult pancreas, adult stomach, appendix, appendix, bile ducts, bowel, buccal mucosa, cecum, colon, colon, colon, colon intestine, colon mucosa, colon-rectum muscularis mucosae epithelium, colonic epithelium, colorectal, deodenum, descending colon, digestive, digestive system epithelium, duodenum, duodenum, duodenum mucosal crypts, esophagus, esophagus, exocrine pancreas, exocrine pancreatic ducts, fetal colon, fetal liver, fetal liver, fetal liver, fundic epithelium, gall bladder, gallbladder, gallbladder bile, gastric, gastric antrum, gastric mucosa, gastrointestinal, gastrointestinal, gastrointestinal epithelia, gastrointestinal epithelium, gastrointestinal tract, gut, hepatic, hepatic endothelia, ileocecum, ileum, ileum, intestinal, intestinal, intestinal brush border, intestinal crypts, intestinal epithelia, intestinal epithelium, intestinal tract, intestine, intestine, intestines, islets, jejunum, jejunum, jejunum brush border, langerhans, large intestine, large intestines, liver, liver, liver skeletal muscle, mouth, mucous acini, non-cancerous liver, normal stomach, omentum, oral cavity, oral epithelia, oral epithelium, oral tongue, palatal epithelia, palatal shelf, palate, pancreas, pancreas acinar ductal epithelium, pancreas islets, pancreatic, pancreatic acini, pancreatic beta-cells, pancreatic duct, pancreatic islets, parotid, parotid, parotid gland, parotid saliva, parotid salivary gland intralobular ducts, rectal, rectum, rectum, salivary, salivary gland, salivary gland, salivary glands, small intestine, small intestine, small intestine, small intestine, small intestines, stomach, stomach wall, sublingual gland, sublingual glands, submandibular, submandibular gland, submaxillary glands, teeth, tongue, tooth, transverse, transverse colon |
| Endocrine | VSIR, BDNF, CHMP1A, FRZB, BIN2, EREG, CASP2, PLXNB3, PRKG1, PEAR1, VAV3, BID, NFATC1, VPS37A, ANGPTL2, DNAJB6, SNAP23, ABHD14B, GMPR2, STX8, ADAMTSL4, PLSCR3 | adrenal, adrenal, adrenal cortex, adrenal gland, adrenal gland, adrenal glands, adrenal glands, adrenal glomerulosa, adrenals, adult placenta, ducts, endocrine glands, fetal adrenal, fetal placenta, gland, glands, hypophysis, hypothalamus, hypothalamus, intestinal glands, lacrimal gland, lateral hypothalamus, parathyroid, parathyroid gland, phaeochromocytoma, pineal gland, pituitary, pituitary gland, pituitary gland, placenta, placenta, placenta, placenta syncytiotrophoblasts, placenta vascular, placenta vascular endothelium, placental, placental, placental endothelium, placental membranes, placental stem villi vessels, placental vascular, placental villi, placental villi, placentas, steroidogenic glands, submucosal gland, submucosal glands, term placenta, thyroid, thyroid, thyroid follicles, thyroid gland, thyroid gland, thyroid glands |
| Integumentary | CCL5, EDAR, SERPINA5, ADAMTSL4 | anagen follicles, basal epidermal layer, basal layer, basal skin layer, club hair, dermal papilla, dermis, eccrine sweat, eccrine sweat glands, exocrine, fetal follicles, fetal skin, follicular, follicular fluid, hair, hair fibers, hair follicle, hair follicles, nail bed epithelium, nail matrix, palmoplantar epidermis, scalp, scalp follicles, scalp skin, scar lesional skin, sebaceous gland, skin, skin epidermis, stratum corneum, stratum granulosum, stratum spinosum, sweat, sweat ducts, sweat gland, sweat gland ducts, sweat glands, sweat glands, upper spinous layers |
| Lymphatic | VSIR, GIPC3, APP, NT5C3A, FKBP1B, SRC, BIN2, TBC1D23, SEPTIN9, CD84, VAV3, BID, CASP8, CCL13, NFATC1, DRG2, DOK2, GTPBP2, PDLIM7, ANGPTL2, CASP3, CA13, DNAJB6, ERBIN, SERPINA5, ABHD14B, CCL17, GMPR2, STX8, ADAMTSL4, PLSCR3 | -rich red pulp, adenoid, adult lymph nodes, beta-cells, bone marrow, fetal spleen, fetal spleen, fetal thymus, fetal thymus, fetal thymus, fetal tonsils, germinal center, germinal centers, hematopoietic, hematopoietic, immune system, lymph, lymph, lymph node, lymph node-containing, lymph nodes, lymphatic, lymphatic vessels, lymphatics, lymphocytic compartment, lymphoid, lymphoid node, lymphoid organs, lymphoid organs, mantle zones, mesenteric lymph nodes, peripheral lymph nodes, peyer patches, peyer's patches, peyers's patches, red pulp, secondary lymphoid, spleen, spleen, thymic medulla, thymus, thymus, thymus epithelium, thymus medulla, tonsil, tonsil, tonsils |
| Musculoskeletal | BDNF, MAP2K6, FRZB, CASP2, TBC1D23, SEPTIN9, VAV3, CASP8, PEAR1, NFATC1, PDLIM7, DRG2, VPS37A, ANGPTL2, CASP3, ENO2, DNAJB6, ERBIN, MAX, SERPINA5, ABHD14B, GMPR2, STX8, ADAMTSL4, PLSCR3, SMAD1 | adult skeletal muscle, appendicular skeleton, articular, articular cartilage, articular cartilages, articular hyaline cartilage, bone, bone matrix, bone-forming sites, bone-forming surfaces, bones, bones, calvaria, calvaria, carpal bones, cartilage, cartilages, cartilaginous, cartilaginous cores, cortical plate, cranial cartilage, deep zone cartilage, dental enamel, dental papilla, dental pulp, dentin, epiphysis, fetal cartilage, fetal perichondrium, ganglia, hip articular cartilage, hypertrophic cartilage, intervertebral disk, invertebral disk, joint capsule, joint cartilage, joints, ligament, ligaments, long bone, long bones, lumbar disk, metaphyseal bone, muscle, muscle, muscle fibers, muscles, osseous, periodontium, rib bone, sarcomeric muscle, skeletal, skeletal muscle, skeletal muscle, skeletal muscle), skeletal muscles, skeletal muscles, spinal muscular, striated muscle, striated muscle, striated muscles, synaptic fibers, synovial, synovial fluid, synovium, tarsal bones, tendon, trabecular bone, vertebrae |
| Nervous | FN1, BDNF, GIPC3, HS6ST1, APP, FKBP1B, FRZB, SRC, CASP2, TBC1D23, SEPTIN9, PLXNB3, PEAR1, VAV3, CASP8, NFATC1, GTPBP2, VPS37A, CASP3, DNAJB6, ERBIN, MAX, LG4, GMPR2, STX8, ADAMTSL4 | adrenal medulla, adult cns, adult nervous central system, amygdala, anterior horn, auerbach plexus, axons, basal ganglia, blood-brain, brain, brain, brain, brain cortex, brain neocortex, brain regions, brain stem, brain structures, brainstem, brainstem, bruch's membrane, caudate nuclei, caudate nucleus, caudate region, central, central nervous, central nervous system, cerebellar nuclei, cerebellum, cerebellum, cerebral cortex, cerebral spinal, cerebro-spinal fluid, choriocapillaris, choroid, choroid plexus, ciliary body, ciliary border, ciliary nonpigmented epithelium, circumvallate papillae, cns, cochlea, cochlea, cone photoreceptors, conjunctival epithelia, conjunctival epithelium, cornea, cornea, corneal, corneal epithelium, corneal stromal layer, corpus callosum, corpus luteum, corpus region, cortex, cortical layers, cranial ganglia, dentate gyrus, dentate nucleus, diencephalon, dorsal root ganglia, dorsal root ganglia, dorsal root ganglion, drg, embryonic retina, extraocular smooth muscle, eye, eye anterior segment, eye lens, eyes, fetal brain, fetal brain, fetal brain, fetal brains, fetal cerebellum, fetal eye, fetal frontal lobe, fetal retinal pigment epithelium, fetal substantia nigra, frontal cortex, frontal lobe, fusiform gyrus, ganglion cell layer, germinal neuroepithelium, globus pallidus, hippocampal ca1, hippocampal dentate gyrus, hippocampal subfields, hippocampus, hippocampus, inner ear, insula, iris, lumbar, medulla, medulla, medulla oblongata, medulla region, midbrain structures, motor cortices, myelinated structures, neocortex, neocortical regions, nerve fiber layer, nervous, nervous system, neural, neural retina, neuroendocrine, neuron, neuronal, neuroretina, neutrophils thyroid gland, nucleus accumbens, occipital, occipital lobe, occipital pole, olfactory bulb, olfactory epithelium, olfactory lobe, olfactory tubercles, ophthalmic nerve, optic nerve, papillary sphincter, parahippocampal cortex, paraolfactory gyri, parietal lobe, parietal lobes, periaxonal myelin, peripheral nerve, peripheral nervous system, peripheral nervous systems, peripheral retina, photoreceptor outer, pigmented epithelium, pns neuroectoderm, pons, pons, posterior perisylvian, postrema, postsynaptic structures, prefrontal cortex, putamen, putamen, retina, retina, retina pigment epithelium, retinal, retinal cone photoreceptors, retinal pericytes, retinal pigment epithelia, retinal pigment epithelium, retinal rod, rod, rod photoreceptors, rolandic area, rostral segment, sclera, spinal chord, spinal chord, spinal cord, spinal cord, spinal cord, spinal cordon, stria vascularis, subiculum, substantia nigra, subthalamic nucleus, sustantia nigra, sympathetic, synaptic fibers, telencephalon, temporal cortex, temporal gyrus, temporal lobe, temporal lobes, thalamus, thalamus, ventral striatum, vertebrae, vestibular system, vestibule |
| Reproductive | VSIR, BDNF, EGF, SKAP1, TBC1D23, SEPTIN9, RAB27B, CASP8, PEAR1, IQGAP2, GTPBP2, ANGPTL2, CASP3, CA13, DNAJB6, SERPINA5, ABHD14B, GMPR2, CCL26, ADAMTSL4, PLSCR3 | adult testis, bartholin's, breast, breast, breast cyst, cerebrum, cervical, cervical squamous epithelium, cervix, cervix, chorion, chorionic villi, decidua, decidua, ectocervical epithelium, embryo testis, endometrium, endometrium, endometrium basalis, endometrium epithelium, epididymis, epididymis, epididymis lumen, excurrent ducts, fallopian tube, fallopian tubes, fallopian tubes, female reproductive, fetal testis, fetal testis, foreskin, genital, genital tract, gingival crevicular, gingival crevicular fluid, gonadal ridge, graaf follicle fluids, isthmus, mammary, mammary epithelia, mammary epithelial cell surfaces, mammary gland, mammary gland, mammary glands, myometrium, neoplastic prostate, nipple aspirate, nipple epidermis, outer myometrial smooth muscle, ovarian, ovarian, ovaries, ovary, ovary, oviduct, penis, prostate, prostate, prostate, prostate epithelium, prostate gland, prostate gland, prostate glands, prostatic, prostrate, reproductive, reproductive system, seminal vesicle, seminal vesicle, seminal vesicles, seminiferous tubules, testes, testicles, testis, testis, umbilical chord, umbilical cord, uterine endometrium, uterine fluid, uterine glandular epithelium, uterine myometrium, uterus, vagina, vaginal epithelium |
| Respiratory | VSIR, BDNF, FRZB, EREG, CASP2, PLXNB3, BID, PEAR1, PRKG1, CCL13, EDAR, GTPBP2, PDLIM7, DRG2, CASP3, DNjAJB6, MAX, ABHD14B, CCL17, STX8, ADAMTSL4, PLSCR3 | airway epithelium, airways, alveolar walls, bronchi, bronchial epithelial, bronchial glands, bronchial submucosal, bronchiolar epithelium, bronchioles, bronchus, bronchus, bronchus-associated, fetal lung, fetal lung, larynx, lung, lung, lung endothelium, lung parenchyma, lung submucosal, lung submucosal gland acinus, lung vascular smooth muscle, lungs, nasal, nasal cavity, nasal mucosa, nasal septal epithelium, nasopharynx, pharynx, pulmonary, pulmonary airways, pulmonary alveoli, respiratory epithelium, respiratory tracts, ribs, trachea, trachea |
| Urinary | EGF, APP, FRZB, EREG, CASP2, TBC1D23, SEPTIN9, PLXNB3, ADAMTS15, PEAR1, ENOX2, EDAR, DRG2, GTPBP2, VPS37A, CASP3, DNAJB6, ERBIN, MAX, SERPINA5, ABHD14B, GMPR2, STX8, ADAMTSL4, PLSCR3 | ascending limbs, bladder, bladder, bladder urothelium, collecting duct, collecting ducts, collecting tubule, convoluted tubule, convoluted tubule, convoluted tubule lumen, convoluted tubules, cortical collecting tubules, descending limbs, distal tubules, fetal bladder, fetal kidney, fetal kidney, fetal kidney, fetal kidneys, glomeruli, glomerulus, henle, kidney, kidney, kidney artery, kidney cortex, kidney distal, kidney glomeruli, kidney medulla, kidneys, mesangium, nephron, nephron segments, non tumor kidney, normal kidney, proximal tubule, proximal tubules, renal proximal tubule, renal proximal tubules, ureter, ureter, urinary bladder, urinary bladder, urogenital, urogenital, urothelium, vas deferens |

Note: The proteins are listed in ascending Bonferroni adjusted P-Value order.

**Supplemental Table 5: Expression NLP Categories by Cell Type for the Top 119 Proteins.**

| Cell Type | Proteins | Keywords |
| --- | --- | --- |
| Adipocyte | C3 | adipocyte, adipocytes |
| Cancer | GIPC3, ENOX2, SERPINA5 | 3, a-431 epidermoid carcinoma, a-549 (lung carcinoma), acute myelocytic leukemia, acute myelogenous leukemia), acute myeloid leukemia, all leukemia/lymphoma lines, bladder cancer, bladder carcinoma, breast cancer, breast cancer lines, breast cancer lines mcf-7, breast cancer lines mda-mb-231, breast cancer lines, breast carcinoma lines, burkitt's lymphoma lines, cancer, cancer lines, carcinoma, carcinoma lines, choriocarcinoma, choriocarcinoma cancer lines, colon adenocarcinoma line t84, colon adenocarcinoma lines, colon cancer lines, colorectal adenocarcinoma line, colorectal cancer, colorectal cancer lines, colorectal tumor, erythroleukemia, erythroleukemia line k-562, fa6, fibrosarcoma, gastric, gastric cancer lines, glioblastoma, glioblastoma lines, glioblastomas, hairy leukemia, hbl-100 breast carcinoma, hel, hematopoietic tumor lines, hepatoular carcinoma, hl-60, hodgkin, hpaf, hs 294t melanoma, ht29-d4 colon carcinoma, imim-pc2, intratumoral nk, k-562, k-562 erythroleukemia, kidney tumor, leiomyomal, leukemia lines, leukemia u-937, leukemia u-937 line, leukemic, leukemic lines, lovo, lung cancer lines, lung carcinoma lines, lung tumor lines, lymphoma, lymphoma lines, malignant, malignant hodgkin lymphoma, malignant melanoma, malignant melanoma lines, mammary carcinoma lines, mcf-7 breast carcinoma, mda-mb-175, mda-mb-435, melanoma, melanoma lines, metastasizing melanoma lines, myelogenous leukemia line kg-1, myelogenous leukemic lines, myeloid leukemia lines, nb4, neoplastic lines, neuro-epithelioma, neuroblastoma, non invasive breast carcinoma lines, non-glial-derived nervous system tumor lines, non-hodgkin lymphoma lines, noneuroblastoma, nonhematopoietic tumor lines, nurse-like, pancreatic cancer lines, pancreatic carcinoma lines, panctu-ii, paraneoplastic tumor, pc-3, promyelocytic leukemia line hl-60, prostate cancer, prostate cancer lines, prostatic adenocarcinoma lines, retinoblastoma lines, several cancer lines, sk-ov-3 (ovary adenocarcinoma), smmc7721, snu-c2b colon carcinoma, sw48, sw480, sw480 colon carcinoma, sw480 colorectal cancer line, testicular tumor, tumor, tumor lines, tumor endothelial, tumor invasive tumors, tumor-derived lines, tumoral, tumorigenic lines, tumors lines, u-251mg, u-937 histiocytic lymphoma lines |
| Chondrocyte | | articular chondrocytes, chondrocyte, chondrocyte-like, chondrocytes, fetal chondrocyte |
| Dendritic | CD84 | cutaneous dendritic, dc, dendritic, follicular dendritic, ikdcs, imddc, immature dendritic, interdigitating reticulum, interferon-producing killer dendritic, mddc, monocyte-derived, monocyte-derived dendritic, myeloid blood dendritic, myeloid dendritic, pdc, pdcs, peripheral blood plasmacytoid dendritic, plasmacytoid, plasmacytoid blood dendritic, plasmacytoid dendritic, plasmacytoids, thymic dendritic, tolerogenic dcs, tonsil dc, tonsil interdigitating dendritic, various dendritic |
| Dental |  | ameloblast, cementoblast, odontoblast, ondotoblasts |
| Endocrine |  | endocrine, enterocyte-like, enterocytes, enteroendocrine, enteroendocrine l, ileal absorptive enterocytes |
| Endothelial | SELP, PEAR1, CRACR2A | angioblasts, aorta endothelial, aortic endothelial, arterial endothelial, artery endothelial, blood brain barrier endothelial, cervical epithelium, umbilical vein endothelial, endiothelial, endothelial, endothelial venules, human umbilical vein endothelial, huvecs, liver sinusoidal endothelial, lung endothelial, lymph vessel endothelial, microvascular capillary endothelial, microvessels endothelial, placenta, umbilical vein endothelial, renal glomeruli endothelial, reticuloendothelial, sinusoidal endothelial, tumor endothelial, umbilical veil endothelial, umbilical vein endothelial, vascular endothelial |
| Epithelial | FN1, TMEM106A, ARL2BP, SDC4 | a-549, airway epithelial, alveolar epithelial, alveolar type 2, alveolar type ii, antral epithelial, atypical epithelial, breast epithelial, breast epithelial line mcf-10a, breast epithelial lines, bronchial, bronchial epithelial, choroid plexus epithelial, ciliary body epithelial, ciliated, colonic, colonic epithelial, columnar epithelial, corneal epithelial, embryonic epithelial, epithelial, epithelial lines, eue, gastric epithelial, gastrointestinal epithelial, hek293, hela, helas3, hep-g2, ht-29 colonic epithelial, intestinal epithelial, intraepithelial cd8-positive t, intraepithelial lymphocytes, kidney epithelial, kidney proximal tubular epithelial, luminal epithelial, lung alveolar type 2, lung epithelial, mammary epithelial, mcf-7, mesothelial, myoepithelial, myoepithelium, nasal, nasal epithelial, nonciliated, paneth, parietal epithelial, pharyngeal epithelial, prostate gland epithelial, renal proximal tubule epithelial, retina pigment epithelial, retinal pigment epithelial, secretory epithelial, small intestinal epithelial, surface epithelial, t-47d, thymic, thymic epithelial, thyrocytes, tracheal surface epithelial, tubular epithelial, type ii alveolar, unpolarized epithelial, vascular epithelial, zr-75-1 |
| Erythrocyte | PEAR1 | erythroblasts, erythrocytes, erythroid, fetal erythrocytes |
| Eye |  | amacrine, cone, photoreceptors, rod photoreceptor |
| Fibroblast | FN1, CHMP1A, LTA4H, HEPH, SDC4 | cerebral pericytes, dermal fibroblasts, fetal fibroblasts, fibroblast, fibroblast lines, fibroblast lines tk, fibroblast-like synoviocytes, fibroblastic, fibroblasts, foreskin fibroblast, foreskin fibroblasts, gingival fibroblasts, mg-63 line, myofibroblasts, pulmonary fibroblasts, skin fibroblasts, stromal fibroblast, stromal fibroblasts, synovial, synovial fibroblasts, synovial fluid |
| Glial | APP, PLXNB3 | astrocytes, astrocytoma, glia, glial, glioma, glioma lines, glioma tissue, liver astrocytes, microglia, microglial, neuro-glial, olgs, oligodendrocytes, oligodendroglia, perivascular astrocytes |
| Granulocyte | CD69, LTA4H, PLXNB3 | basophil, basophils, bone marrow neutrophils, eosinophils, granular, granule, granulocyte, granulocytes, granulocytic, granulocytic lineage, inflammatory, neutrophil, neutrophil lineage, neutrophils, peripheral blood basophils, peripheral blood granulocytes, peripheral blood neutrophils, polynuclear neutrophils |
| Hematopoietic | | hematopoetic, hematopoietic, hematopoietic lines, hematopoietic lineage, hematopoietic precursors, hematopoietic progenitor, hematopoietic stem, hemopoietic |
| Kidney | TMEM106A, PLXNB3 | bowman's capsule, distal tubular, embryonal kidney, embryonic kidney, glomerular epithelium, glomerular mesangial, hsc, interstitial, kidney distal tubular, mesangial, podocyte, podocytes, proximal tubule, renal, renal lines, renal proximal tubular, tubular |
| Leukocytes Nyd | SKAP1, EREG, CD84, HEPH, PEAR1, NFATC1, PDLIM7, DRG2, DNAJB6, ABHD14B, GMPR2, ADAMTSL4 | ag-presenting, apcs, blood leukocytes, blood mononuclear, bone, bone marrow, bone marrow mononuclear, bone marrow-derived, bone marrow-derived mesenchymal stem, bone trabecular, bone-derived, cortical thymocytes, flattened bone-lining, immune, immunoblasts, immunocyte lines, interstitial leukocytes, leukocyte, leukocyte lines, leukocytes, mast, mononuclear, mononuclear leukocytes, myeloblast, normal mast, pbmc, pbmcs, peripheral blood leukocyte, peripheral blood leukocytes, peripheral blood mononuclear, peripheral blood mononuclear leukocytes, peripheral leukocyte, peripheral leukocytes, peripheral mononuclear, phagocytes, phagocytic, plasma, pmns, polymorphonuclear leukocytes, polynuclear, promyelocyte stage, promyelocytes, promyelocytic, submucosal leukocytes, thymocytes, urothelial, white blood |
| Liver | FN1 | crypt, hepatic, hepatic parenchymal, hepatic stellate, hepatocytes, hepatoma, hepatoma lines, liver hepatocytes |
| Lymphocyte | VSIR, SKAP1, APP, CD69, BIN2, LTA4H, CD84, CCL5, NFATC1, CD226, DGKA, PLSCR3, CRACR2A | -differentiated hl-60, alpha-beta t, b, b lineage, b-, b- lineage, b- lines, b--like line raji, b-1, b-lymphocyte, b-lymphocytes, b-lymphoid lines, b-lymphomas, blood lymphocytes, ca4, cytolytic, cytotoxic t lymphocytes, cytotoxic t-lymphocytes, decidual nk, effector, epstein-barr virus-transformed lymphoblastoid lines, fetal nk-, gamma delta t, gamma-delta t, gamma-delta t-, germinal center, germinal center b-, germinal centers, group2 innate lymphoid, helper t-, hsb, hut 78, ilc2s, intraepithelial lymphocytes, intratumoral nk, jurkat, jurkat lines, jurkat t- leukemia, jurkat t- line, large lymphocytes, lymphoblast, lymphoblasts, lymphocyte, lymphocytes, lymphocytic lines, lymphocytic lineage, lymphoid, lymphoid lines, lymphoid organs, marginal zone b-, mature b, melanoma-specific cytotoxic t clones, memory b-, memory gamma-delta t, memory t-, memory th17, molt-4, molt-4 lines, naive t, natural killer, natural killer lines nkl, natural killer (nk), neoplastic b- and t- lines, neoplastic b- and t- lines, nk, nk subsets, nk 62, nk-, nk- line, nkt, normal germinal center (gc) b-, pbl, peripheral blood lymphocyte, peripheral blood lymphocytes, peripheral blood memory t-, peripheral blood t-, peripheral blood t-lymphocytes, peripheral lymphocytes, peripheral memory, peripheral t-, peripherical blood lymphocytes, plasma b-, pre b-, pre t-, pre-b-, pro-b precursors, raji b-lymphoblasts, reed-sternberg (hrs), sup-t1, t, t populations, t lymphocytes, t-, t- clones, t- leukemia lines molt-4, t- lineage, t- lines, t- lines harris, t- lymphoid lines, t- subsets, t-helper, t-helper 2, t-lymphoblasts, t-lymphocytes, th0, th1, thymus-derived t-, tonsillar germinal center centrocytes, transitional b, treg, treg), yt |
| Macrophage | CD69, CD84, PEAR1, CCL5 | -macrophage, a, alveolar macrophages, bone marrow macrophages, cd68, cortical macrophages, decidual macrophages, epidermal langerhans, epidermoid, hofbauer, kg-1, kupffer, langerhans, langerhans', liver kupffer, liver kupffer, lung alveolar macrophages, m1 macrophages, macrophage, macrophage line, macrophage lines u-937, macrophage progenitor, macrophage-like, macrophages, meningeal macrophages, monocyte-derived macrophages, monocyte-derived macrophages, non sec-, perivascular macrophages, placental macrophages, red pulp macrophages, spleen macrophage, tissue macrophages |
| Monocytes | VSIR, LTA4H, CD84 | apcs monocytes, cd11b monocytes, mono-mac-6, monocyte, monocyte-like line u-937, monocyte-related, monocytes, monocytic, monocytic lines, myelomonocytic, myelomonocytic lineage, peripheral blood monocytes, peripheral monocytes, promonocytic, thp-1, thp-1 monocytes |
| Mucous |  | bronchial goblet, ciliated bronchiolar, goblet, intestinal mucosa, mucous, mucus, mucus-secreting, nasal goblet secretory, upper gastric mucosal |
| Muscle | PEAR1, CCL13 | airway smooth muscle, aortic smooth muscle, arterial smooth muscle, artery smooth muscle, cardiac, cardiac myocytes, cardiomyocytes, cerebral artery smooth muscle, coronary muscle, muscle, myoblasts, myocytes, myotubes, perivascular, placental vascular smooth muscle, placental villi smooth muscle, pulmonary artery smooth muscle, skeletal muscle, smooth, smooth muscle, umbilical vein smooth muscle, vascular, vascular smooth, vascular smooth muscle, vascular wall, vsmc |
| Myeloid | VSIR, GIPC3 | chronic myelogenous, early myeloid lines, monocytic/myeloid lineage, mucosal myeloid, myeloid, myeloid lines, myeloid lineage, myeloid lineages, myeloid progenitor, myeloid-derived suppressor, myeloids, myeloma, myeloma line u266b1, myeloma line u266r |
| Neuron | SRC, PLXNB3, PEAR1, CASP3, ENO2 | axons, basket, brain neurons, ca2, central neurons, cerebellar purkinje, cerebral cortex, cortical neurons, dentate gyrus granule neurons, dopaminergic neurons, dorsal root ganglia neurons, dorsal root ganglion, gabaergic neurons, granule neurons, gray matter neurons, hippocampal pyramidal neurons, hippocampus pyramidal, hippocampus pyramidal neurons, lines, motoneurons, neocortical neurons, neural, neural crest, neural progenitor, neural stem, neuroendocrine, neuroendocrine epithelium, neuron, neuronal, neurons, olfactory receptor neurons, peripheral, peripheral neurons, pontine nuclei, purkinje, purkinje neurons, pyramidal, pyramidal neurons, retinal ganglion, schwann, schwann culture, somatomotor, spinal cord neurons, stellate, striatal neurons |
| Non-Hematopoietic | | non-hematopoetic, non-hematopoietic, nonhematopoietic |
| Osteoblast | CHMP1A, SRC, PEAR1 | bone osteoblasts, giant osteoclast-like, osteoblast, osteoblast line mg-63, osteoblast line saos-2, osteoblast-like, osteoblasts, osteoclasts, osteocytes, osteogenic, osteosarcoma lines, primary ossification center-associated, subchondral bone osteoblasts |
| Other | APP | epidermal basal, inner ear hair, intimal, large t sv40 antigen-, non-immune, non-neuronal, nonmuscle, normal lines, populations, villous |
| Other Blood | NT5C3A, LTA4H | blood, blood lines, cord blood, peripheral blood, peripherical blood, red blood, reticulocytes |
| Pancreatic |  | beta-, betaacinar, ductile, islet, pancreas islet beta, pancreatic, pancreatic lines, pancreatic acinar, pancreatic beta, pancreatic islet |
| Platelet | GP6, GP5, SELP, D69, TREML1, SRC, LTA4H, CD84, PEAR1, VAMP8, SERPINA5 | eosinophil platelets, megakaryoblastic, megakaryocytes, megakaryocytic, platelet, platelets, thrombocytes |
| Reproductive | DNAJB6, SERPINA5 | cumulus, cyto- and syncytiotrophoblastic, cytotrophoblasts, endometrial glandular, extravillous trophoblast, germ, gonocytes, granulosa, interstitial leydig, leydig, luteinized granulosa, migratory primordial germ, myometrial, oocytes, ovarian granulosa, ovary, post-meiotic, postnatal leydig, secretory endometrial, sertoli, spermatocytes, spermatogenic, spermatogonia, spermatogonias, spermatozoa, syncytiotrophoblast, syncytiotrophoblasts, testicular somatic, trophoblast, trophoblasts, x |
| Secretory | SELP | chief, chromaffin, clara, enterochromaffin, fundic, gastric parietal, secretory, serous-like, weibel-palade bodies, zymogen-producing |
| Skin | VAV3, EDAR, SERPINA5 | basal keratinocytes, cornified, epidermal basal layer keratinocytes, epidermal keratinocytes, keratinocyte, keratinocytes, megakaryocytic lines, melanocyte, melanocytes, pigment, skin keratinocytes, suprabasal keratinocytes |
| Spleen | SKAP1 | fetal spleen, spleen, splenic marginal zone, splenocytes |
| Stem |  | cambial, cml) stem, embryonic stem, esc, mesenchymal, mesenchymal stem, stem |
| Stromal |  | bone marrow stromal, endometrial stromal, non-cancerous stromal, stromal, stromal type |

Note: The proteins are listed in ascending Bonferroni adjusted P-Value order. Keywords and cell types are generated from all proteins with available information. The table displays the top 119 proteins. Some cell types listed were not linked to any of the 119 proteins (eg. Pancreatic).

**Additional file 1: Figure Legend**

**Additional file 1: Fig. S1. Recursive Feature Selection of 119 Protein Results after 10000 Runs** The plot showcases the number of Recursive Feature Selection runs each protein made it into the top 10. The 5000 run threshold(50%) is shown with a green dashed line and the 8000 run threshold (80%) is shown with an orange dashed line. Out of the 119 proteins, only the shown molecules made it into the top 10 at least once.

**Additional file 1: Fig. S1.**

**
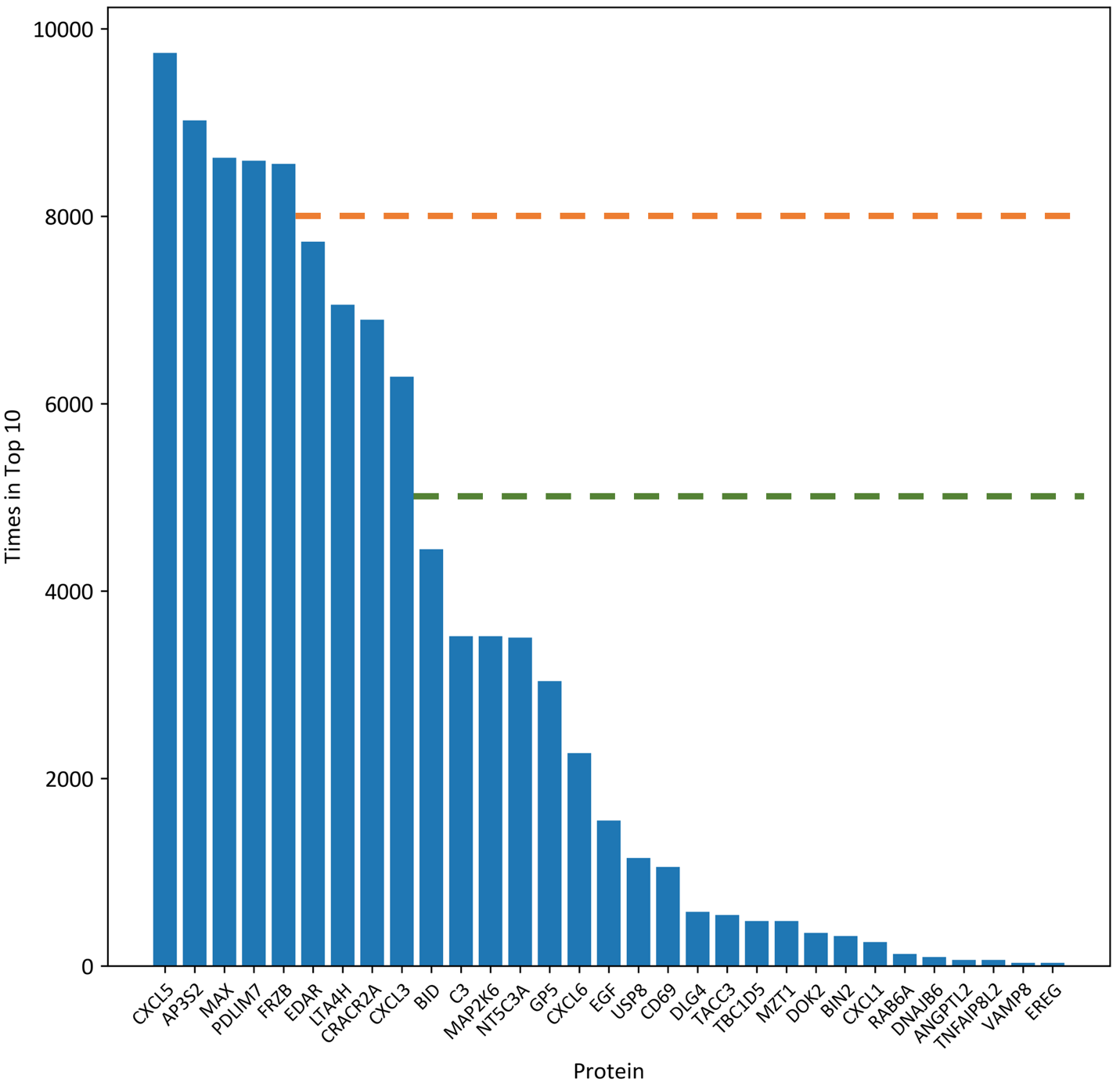
**

**References**

1. Chang MS, Mcninch J, Basu R, Simonet S. (1994) Cloning and characterization of the human neutrophil-activating peptide (ENA-78) gene. *Journal of Biological Chemistry* **269:** 25277-25282.

2. Li A*, et al.* (2011) Overexpression of CXCL5 Is Associated With Poor Survival in Patients With Pancreatic Cancer. *The American Journal of Pathology* **178:** 1340-1349.

3. Strieter RM, Belperio JA, Keane MP. (2002) CXC Chemokines in Angiogenesis Related to Pulmonary Fibrosis*. *Chest* **122:** 298S-301S.

4. Kawamura M*, et al.* (2012) CXCL5, a promoter of cell proliferation, migration and invasion, is a novel serum prognostic marker in patients with colorectal cancer. *European Journal of Cancer* **48:** 2244-2251.

5. Begley LA*, et al.* (2008) CXCL5 Promotes Prostate Cancer Progression. *Neoplasia* **10:** 244-254.

6. Goodman RB*, et al.* (1996) Inflammatory cytokines in patients with persistence of the acute respiratory distress syndrome. *American journal of respiratory and critical care medicine* **154:** 602-611.

7. Buckland J. (2014) Citrullination alters the inflammatory properties of chemokines in inflammatory arthritis. *Nature Reviews Rheumatology* **10:** 446-446.

8. Zwiewka M*, et al.* (2011) The AP-3 adaptor complex is required for vacuolar function in Arabidopsis. *Cell Research* **21:** 1711-1722.

9. Dell’Angelica EC. (2009) AP-3-dependent trafficking and disease: the first decade. *Current Opinion in Cell Biology* **21:** 552-559.

10. Grabner CP, Price SD, Lysakowski A, Cahill AL, Fox AP. (2006) Regulation of large dense-core vesicle volume and neurotransmitter content mediated by adaptor protein 3. *Proc Natl Acad Sci U S A* **103:** 10035-10040.

11. Kazakova E*, et al.* (2017) Gas6 gene rs8191974 and Ap3s2 gene rs2028299 are associated with type 2 diabetes in the northern Chinese Han population. *Acta Biochimica Polonica* **64**.

12. Kooner JS*, et al.* (2011) Genome-wide association study in individuals of South Asian ancestry identifies six new type 2 diabetes susceptibility loci. *Nature Genetics* **43:** 984-989.

13. Mohlke KL, Boehnke M. (2015) Recent advances in understanding the genetic architecture of type 2 diabetes. *Human Molecular Genetics* **24:** R85-R92.

14. Guardia CM, De Pace R, Mattera R, Bonifacino JS. (2018) Neuronal functions of adaptor complexes involved in protein sorting. *Current Opinion in Neurobiology* **51:** 103-110.

15. Wagner AJ, Le Beau MM, Diaz MO, Hay N. (1992) Expression, regulation, and chromosomal localization of the Max gene. *Proceedings of the National Academy of Sciences* **89:** 3111-3115.

16. Carabet L, Rennie P, Cherkasov A. (2018) Therapeutic Inhibition of Myc in Cancer. Structural Bases and Computer-Aided Drug Discovery Approaches. *International Journal of Molecular Sciences* **20:** 120.

17. Comino-Méndez I*, et al.* (2011) Exome sequencing identifies MAX mutations as a cause of hereditary pheochromocytoma. *Nature Genetics* **43:** 663-667.

18. Burnichon N*, et al.* (2012) MAX Mutations Cause Hereditary and Sporadic Pheochromocytoma and Paraganglioma. *Clinical Cancer Research* **18:** 2828-2837.

19. Blaževitš O*, et al.* (2020) MYC-Associated Factor MAX is a Regulator of the Circadian Clock. *International Journal of Molecular Sciences* **21:** 2294.

20. Guy PM, Kenny DA, Gill GN. (1999) The PDZ Domain of the LIM Protein Enigma Binds to β-Tropomyosin. *Molecular Biology of the Cell* **10:** 1973-1984.

21. Krcmery J*, et al.* (2013) Loss of the Cytoskeletal Protein Pdlim7 Predisposes Mice to Heart Defects and Hemostatic Dysfunction. *PLoS ONE* **8:** e80809.

22. Perisic Matic L*, et al.* (2016) Phenotypic Modulation of Smooth Muscle Cells in Atherosclerosis Is Associated With Downregulation of LMOD1, SYNPO2, PDLIM7, PLN , and SYNM. *Arteriosclerosis, Thrombosis, and Vascular Biology* **36:** 1947-1961.

23. Li A, Ponten F, dos Remedios CG. (2012) The interactome of LIM domain proteins: The contributions of LIM domain proteins to heart failure and heart development. *Proteomics (Weinheim)* **12:** 203-225.

24. D'Cruz R*, et al.* (2016) PDLIM7 is a novel target of the ubiquitin ligase Nedd4-1 in skeletal muscle. *Biochemical Journal* **473:** 267-276.

25. Bilodeau PA, Coyne ES, Wing SS. (2016) The ubiquitin proteasome system in atrophying skeletal muscle: roles and regulation. *Am J Physiol Cell Physiol* **311:** C392-403.

26. Kowalczyk-Quintas C, Schneider P. (2014) Ectodysplasin A (EDA) – EDA receptor signalling and its pharmacological modulation. *Cytokine & Growth Factor Reviews* **25:** 195-203.

27. Mikkola ML, Thesleff I. (2003) Ectodysplasin signaling in development. *Cytokine & Growth Factor Reviews* **14:** 211-224.

28. Cluzeau C*, et al.* (2011) Only four genes (EDA1, EDAR, EDARADD, and WNT10A) account for 90% of hypohidrotic/anhidrotic ectodermal dysplasia cases. *Human Mutation* **32:** 70-72.

29. Laurikkala J*, et al.* (2002) Regulation of hair follicle development by the TNF signal ectodysplasin and its receptor Edar. *Development* **129:** 2541-2553.

30. Fujimoto A*, et al.* (2008) A scan for genetic determinants of human hair morphology: EDAR is associated with Asian hair thickness. *Human Molecular Genetics* **17:** 835-843.

31. Rudberg PC, Tholander F, Andberg M, Thunnissen MMGM, Haeggström JZ. (2004) Leukotriene A4 Hydrolase. *Journal of Biological Chemistry* **279:** 27376-27382.

32. Fourie AM. (2009) Modulation of inflammatory disease by inhibitors of leukotriene A4 hydrolase. *Curr Opin Investig Drugs* **10:** 1173-1182.

33. Grice CA, Gomez L. (2008) Current status of leukotriene A4 hydrolase inhibitors. *Expert Opinion on Therapeutic Patents* **18:** 1333-1350.

34. Vo TTL, Jang WJ, Jeong CH. (2018) Leukotriene A4 hydrolase: an emerging target of natural products for cancer chemoprevention and chemotherapy. *Annals of the New York Academy of Sciences* **1431:** 3-13.

35. Srikanth S*, et al.* (2010) A novel EF-hand protein, CRACR2A, is a cytosolic Ca2+ sensor that stabilizes CRAC channels in T cells. *Nature Cell Biology* **12:** 436-446.

36. Srikanth S*, et al.* (2016) A large Rab GTPase encoded by CRACR2A is a component of subsynaptic vesicles that transmit T cell activation signals. *Science signaling* **9:** ra31-ra31.

37. Wu B*, et al.* (2021) Biallelic mutations in calcium release activated channel regulator 2A (CRACR2A) cause a primary immunodeficiency disorder. *eLife* **10**.

38. Notarangelo LD, Bacchetta R, Casanova J-L, Su HC. (2020) Human inborn errors of immunity: An expanding universe. *Science Immunology* **5:** eabb1662.

39. Gulati K*, et al.* (2018) Molecular cloning and biophysical characterization of CXCL3 chemokine. *International Journal of Biological Macromolecules* **107:** 575-584.

40. Laing KJ, Secombes CJ. (2004) Chemokines. *Developmental & Comparative Immunology* **28:** 443-460.

41. Kusuyama J, Komorizono A, Bandow K, Ohnishi T, Matsuguchi T. (2016) CXCL3 positively regulates adipogenic differentiation. *Journal of Lipid Research* **57:** 1806-1820.

42. Guan J*, et al.* (2021) Clinical significance and biological functions of chemokine CXCL3 in head and neck squamous cell carcinoma. *Bioscience Reports* **41**.

43. Gui S-L*, et al.* (2016) Overexpression of CXCL3 can enhance the oncogenic potential of prostate cancer. *International Urology and Nephrology* **48:** 701-709.

44. Vicari AP, Caux C. (2002) Chemokines in cancer. *Cytokine & Growth Factor Reviews* **13:** 143-154.

45. Leyns L, Bouwmeester T, Kim S-H, Piccolo S, De Robertis EM. (1997) Frzb-1 Is a Secreted Antagonist of Wnt Signaling Expressed in the Spemann Organizer. *Cell* **88:** 747-756.

46. Dale CT. (1998) Signal transduction by the Wnt family of ligands. *Biochemical Journal* **329:** 209-223.

47. Jang M-H*, et al.* (2013) Secreted Frizzled-Related Protein 3 Regulates Activity-Dependent Adult Hippocampal Neurogenesis. *Cell Stem Cell* **12:** 215-223.

48. Rich CA*, et al.* (2018) Olfactory ensheathing cells abutting the embryonic olfactory bulb express Frzb , whose deletion disrupts olfactory axon targeting. *Glia* **66:** 2617-2631.

49. Guo Y*, et al.* (2008) Frzb, a Secreted Wnt Antagonist, Decreases Growth and Invasiveness of Fibrosarcoma Cells Associated with Inhibition of Met Signaling. *Cancer Research* **68:** 3350-3360.

50. Rodriguez-Lopez J, Pombo-Suarez M, Liz M, Gomez-Reino JJ, Gonzalez A. (2007) Further evidence of the role of frizzled-related protein gene polymorphisms in osteoarthritis. *Annals of the Rheumatic Diseases* **66:** 1052-1055.

51. Gao G*, et al.* (2010) No association of the polymorphisms of the frizzled-related protein gene with peak bone mineral density in Chinese nuclear families. *BMC Medical Genetics* **11:** 1.

52. Lories RJ, Boonen S, Peeters J, De Vlam K, Luyten FP. (2006) Evidence for a differential association of the Arg200Trp single-nucleotide polymorphism in FRZB with hip osteoarthritis and osteoporosis. *Rheumatology* **45:** 113-114.

53. Leijten JC*, et al.* (2013) GREM1, FRZB and DKK1 mRNA levels correlate with osteoarthritis and are regulated by osteoarthritis-associated factors. *Arthritis Research & Therapy* **15:** R126.
